# Comparative microRNA profiling of *Trypanosoma cruzi* infected human cells

**DOI:** 10.3389/fcimb.2023.1187375

**Published:** 2023-06-21

**Authors:** Natalia Rego, María Gabriela Libisch, Carlos Rovira, Juan Pablo Tosar, Carlos Robello

**Affiliations:** ^1^ Unidad de Bioinformática, Institut Pasteur de Montevideo, Montevideo, Uruguay; ^2^ Laboratorio de Genómica Evolutiva, Facultad de Ciencias, Universidad de la República, Montevideo, Uruguay; ^3^ Laboratorio de Interacciones Hospedero Patógeno/UBM, Institut Pasteur de Montevideo, Montevideo, Uruguay; ^4^ Department of Clinical Sciences Lund, Division of Oncology, Lund University, Lund, Sweden; ^5^ Laboratorio de Genómica Funcional, Institut Pasteur de Montevideo, Montevideo, Uruguay; ^6^ Unidad de Bioquímica Analítica, Centro de Investigaciones Nucleares, Facultad de Ciencias, Universidad de la República, Montevideo, Uruguay; ^7^ Departamento de Bioquímica, Facultad de Medicina, Universidad de la República, Montevideo, Uruguay

**Keywords:** microRNAs, *Trypanosoma cruzi*, cardiomyocytes, epithelial cells, macrophages, host-parasite interaction, post-transcriptional regulation

## Abstract

**Introduction:**

*Trypanosoma cruzi*, the causative agent of Chagas disease, can infect almost any nucleated cell in the mammalian host. Although previous studies have described the transcriptomic changes that occur in host cells during parasite infection, the understanding of the role of post-transcriptional regulation in this process is limited. MicroRNAs, a class of short non-coding RNAs, are key players in regulating gene expression at the post-transcriptional level, and their involvement in the host-*T. cruzi* interplay is a growing area of research. However, to our knowledge, there are no comparative studies on the microRNA changes that occur in different cell types in response to *T. cruzi* infection.

**Methods and results:**

Here we investigated microRNA changes in epithelial cells, cardiomyocytes and macrophages infected with *T. cruzi* for 24 hours, using small RNA sequencing followed by careful bioinformatics analysis. We show that, although microRNAs are highly cell type-specific, a signature of three microRNAs -miR-146a, miR-708 and miR-1246, emerges as consistently responsive to *T. cruzi* infection across representative human cell types. *T. cruzi* lacks canonical microRNA-induced silencing mechanisms and we confirm that it does not produce any small RNA that mimics known host microRNAs. We found that macrophages show a broad response to parasite infection, while microRNA changes in epithelial and cardiomyocytes are modest. Complementary data indicated that cardiomyocyte response may be greater at early time points of infection.

**Conclusions:**

Our findings emphasize the significance of considering microRNA changes at the cellular level and complement previous studies conducted at higher organizational levels, such as heart samples. While miR-146a has been previously implicated in *T. cruzi* infection, similarly to its involvement in many other immunological responses, miR-1246 and miR-708 are demonstrated here for the first time. Given their expression in multiple cell types, we anticipate our work as a starting point for future investigations into their role in the post-transcriptional regulation of *T. cruzi* infected cells and their potential as biomarkers for Chagas disease.

## 1 Introduction

Chagas disease (CD), or American trypanosomiasis, is caused by the kinetoplastid parasite *Trypanosoma cruzi* (Chagas, 1909). Recognized as a neglected tropical disease (NTD) since 2005, CD primarily affects about 6-7 million individuals, mostly in Latin America (Bonney, 2014; Pérez-Molina and Molina, 2018; WHO, 2022). In the mammalian host, *T. cruzi* infective and non-replicative trypomastigotes are, unlike other intracellular parasites, capable of infecting almost any nucleated cell (Fernandes and Andrews, 2012). Upon attaching to host cells, trypomastigotes are internalized within a parasitophorous vacuole (PV), a membrane-bound compartment comprised of host cell endolysosomal system components (Andrade and Andrews, 2005; Batista et al., 2020). The transition to amastigotes, the replicative form, encompasses the disruption of the PV and the subsequent escape to the cytosol. Reports show that PV disruption occurs at 12 to 15 hours post-infection (hpi), depending upon host cell type and parasite strain (Stecconi-Silva et al., 2003). After cycles of binary division for 3-5 days, amastigotes differentiate into bloodstream trypomastigotes that are released upon rupture of the cell membrane to infect neighboring cells or disseminate through the blood to other locations in the body. Previously we have studied the host cell transcriptomic changes that follow *T. cruzi* infection (Dm28c strain) at early and intermediate time points and described the different responses that cardiomyocytes, epithelial cells (HeLa) and macrophages (THP1) exhibit (Chiribao et al., 2014; Libisch et al., 2018; Libisch, 2020). We found that *T. cruzi* reprograms human cells, which probably contributes to the generation of favorable environments for the infection. Epithelial cells constitute the first barrier for the establishment of infection while macrophages are responsible for host defenses and cardiomyocytes are involved in one of the most important consequences of CD, chronic Chagas cardiomyopathy (CCC).

Together with other small RNAs, microRNAs (miRNAs), a class of short (21–23 nt) non-coding RNAs, have pivotal roles in the post-transcriptional regulation of gene expression. They inhibit specific mRNAs by binding to complementary sequences, usually located in the 3′ UTR, leading to mRNA destabilization and a reduction in their translation output (Ambros, 2004; Bartel, 2004; Bartel, 2018). miRNAs have key roles in a wide range of biological processes, and dysregulation of miRNA expression has been implicated in numerous disorders, including cancer and infectious diseases (Dalmay and Edwards, 2006; Leung and Sharp, 2010; Smith-Vikos and Slack, 2012; de Lucia et al., 2017; Chandan et al., 2020). There is considerable interest in the use of altered miRNAs signatures as biomarkers and in miRNAs as therapeutic bases (Slota and Booth, 2019). As players in host-parasite interactions, they are ideally suited because they are non-antigenic (Umbach and Cullen, 2009) and mediate gene regulation with speed and reversibility (Hobert, 2008). In the host cell miRNAs can favour the elimination of the pathogen (Chen et al., 2007) but the intracellular parasite may, in turn, modulate its own and host miRNAs, promoting a subversion strategy and survival (Lemaire et al., 2013; Zheng et al., 2013; Aguilar et al., 2019). Besides, miRNAs and other small RNAs delivered by extracellular vesicles are particularly interesting as putative mediators of inter-kingdom communication (Stanton, 2021). Thus, the role of miRNAs, among other non-coding RNAs, in the parasite-host interplay has gained growing interest (Manzano-Román and Siles-Lucas, 2012; Zheng et al., 2013; Linhares-Lacerda and Morrot, 2016; Bayer-Santos et al., 2017; Acuña et al., 2020; Paul et al., 2020; Rojas-Pirela et al., 2022).

As in the rest of trypanosomatids, *T. cruzi* lacks a canonical miRNA-induced silencing mechanism (Lye et al., 2010) but it is known to express other small non-coding RNAs (sncRNAs)(Garcia-Silva et al., 2010; Franzén et al., 2011; Bayer-Santos et al., 2014). Indeed, sncRNAs can be delivered by extracellular vesicles into HeLa cells, inducing large gene expression changes (Garcia-Silva et al., 2014). Not surprisingly, evidence shows that *T. cruzi* triggers changes in the expression of miRNAs in the infected cells (Linhares-Lacerda and Morrot, 2016; Bayer-Santos et al., 2017; Acuña et al., 2020; Oliveira et al., 2020; Paul et al., 2020; Rojas-Pirela et al., 2022). Due to the clinical relevance of CCC, most of the studies of miRNA changes in *T. cruzi* infection used mouse models of CCC and/or patient samples (Ferreira et al., 2014; Monteiro et al., 2015; Navarro et al., 2015; Ferreira et al., 2017; Linhares-Lacerda et al., 2018; Nonaka et al., 2019; Nonaka et al., 2021). Further, miRNA changes were also studied in thymic epithelial cells (Linhares-Lacerda et al., 2015) and human placental explants (Medina et al., 2020), in relation with CD thymic atrophy and congenital transmission, respectively. Some miRNAs, such as the immunomiRs miR-21 and miR-146a (Kroesen et al., 2015), turned repeatedly upregulated during *T. cruzi* infection. Others, such as miR-322, appear to have a dichotomous role (miR-322 was downregulated and upregulated in cardiac and thymic tissue, respectively). Comparison and interpretation of these limited studies is hampered by the diversity of models and experimental designs involved (Libisch et al., 2021).

In this work, we aimed to expand our knowledge about miRNA changes in host cells infected with *T. cruzi* parasites. While miRNAs tend to have highly cell type specific expression profiles (Ludwig et al., 2016; Patil et al., 2022), we wondered whether *T. cruzi* exhibits a shared signature when modeling the host environment at the miRNA level. We infected three cell types of human origin: cardiomyocytes, epithelial cells (HeLa) and macrophages (THP1-derived), with Dm28c strain parasites (Contreras et al., 1985; Contreras et al., 1988), and analyzed miRNA expression levels at 24 hpi, in the amastigote replicative phase (Stecconi-Silva et al., 2003; Andrade and Andrews, 2005). We used a dual small RNA-seq approach to obtain sequences from both host and parasites (Westermann et al., 2012; Westermann et al., 2017). Thus, we were able to investigate not only the host cell miRNA landscape but also whether the parasites contributed with miRNA-like species, focusing on small RNAs which could mimic known human miRNAs. In the case of cardiomyocytes, our main results were complemented with dual small RNA-seq data obtained at early infection times (0 and 3 hpi). Our results suggest a trio of host miRNAs (miR-1246, miR-146a and miR-708) as consistently responsive to *T. cruzi* infection across these representative cell-types. Besides, we did not find any miRNA-like mimic derived from the parasites.

## 2 Materials and methods

### 2.1 Cell culture, parasites, and infection assays

HeLa human cells were maintained in Dulbecco’s Modified Eagle’s Medium (DMEM) (Gibco, USA) and the THP1 cells in RPMI-1640 (Gibco, USA) with GlutaMax (Gibco, USA); both cell lines were supplemented with 10% (v/v) heat inactivated fetal bovine serum (FBS) at 37°C in a 5% CO2 atmosphere. The THP1 monocyte cells were differentiated to macrophages using PMA (phorbol-12-myristate-13-acetate; Sigma-Aldrich, USA) 200 nM for 48 h. Then the cells were washed and grown in RPMI-10% FBS, for 24 h before infection experiments. Human primary cardiomyocytes (Celprogen, USA) derived from adult cardiac tissue were grown in Human Cardiomyocyte Primary Cell Culture medium (Celprogen, USA) supplemented with 10% (v/v) heat inactivated FBS at 37°C in a 5% CO2 atmosphere. Dm28c strain was used throughout this work (Contreras et al., 1985). This strain is classified as TcI according to the discrete typing unit system (Zingales et al., 2012), and as clade A according to a recently proposed phylogenetic-based nomenclature (Berná et al., 2021). For infection assays, semi-confluent cells were incubated with Vero cell-derived trypomastigotes (10:1 parasite:cell ratio), centrifuged for 5 min at 1500 x g to synchronize adhesion, and allowed to invade for 2 h. After the interaction period, parasites were removed, and cells were washed five times with PBS and incubated with the corresponding medium. Infected and control samples were taken at 24 h after the interaction period (24 hpi). To assess early host miRNA responses in cardiomyocytes, both infected and control samples were collected at 0 and 3 h after interaction. The interaction time between parasites and cardiomyocytes was mantained for 2 h.

The percentage of infected cells was obtained at 24 hpi by visualizing DAPI-stained cells by fluorescent microscopy (Thermo Fisher Scientific, USA). Due to the low number of intracellular parasites at 24 hpi, particularly in the THP1-derived monocyte cultures, the infection index (Aoki et al., 2020) was calculated at 72 hpi.

### 2.2 Library preparation and sequencing

Total RNA was isolated with Direct-zol RNA MiniPrep kit (Zymo, USA), which retained RNA ≥17 nt, as described by the manufacturer, and treated with RNAse-free DNAse to remove contaminating DNA. Total RNA was quantified with the Qubit RNA HS Assay Kit (Thermo Fisher Scientific, USA) and the quality was assessed with Agilent 2100 Bioanalyzer (Agilent Technologies, USA); only samples with RIN above 7 were used. The libraries were constructed using the NEBNext Multiplex Small RNA Library Prep set for Illumina kit (New England Biolabs, USA). After an amplification step of 12 PCR cycles the miRNA-corresponding bands were cut and purified. Sample size distribution was determined using the Bioanalyzer (Agilent Technologies, USA) with the Agilent High Sensitivity DNA Kit (Agilent Technologies, USA). Libraries were sequenced with 50 bp single-end reads on an Illumina MiSeq (Illumina, USA).

### 2.3 Pre-processing of raw sequencing reads

An average of 6.7 million raw reads per sample were obtained (NCBI’s accession PRJNA607998 and **Table S1**). Sequences matching the 3’ adaptor sequence 5’-AGATCGGAAGAGCACACGTCT-3’) were identified and trimmed with cutadapt v1.14 (Martin, 2011). A minimum matching of the 6 first bases of the adaptor sequence was required as well as final read lengths of 18 to 44 bases. The R Bioconductor package ShortRead v1.50.0 (Morgan et al., 2009) and its function dustyFilter were used to further discard low complexity reads (threshold=20). After these trimming and filtering steps, an average of 4.8 million clean, short reads per sample were obtained. Sequence quality of raw and processed reads was assessed with FastQC v0.11.9 and MultiQC v1.11 (Andrews, 2010; Ewels et al., 2016).

### 2.4 Small RNA-seq alignment

Given the dual transcriptome nature of the experimental approach, reads were mapped to a joint dual-genome composed by the human reference (build GRCh38/hg38 from https://genome.ucsc.edu/, September 2018) and the *T. cruzi* Dm28c genome (Berná et al., 2018). Sequences were aligned using bowtie v1.2.2 (Langmead et al., 2009), allowing one mismatch (-v 1), and reported all best alignments for reads that mapped equally well to more than one genomic location (‐a ‐‐best ‐‐strata). Reads with more than 50 possible alignments (-m 50) were suppressed. Samtools v1.13 (Li et al., 2009) and custom scripts were used to split the reads into three categories: “hg38”, “Dm28c” and dual “hg38-Dm28c”, for those reads aligning only to hg38 genome, only to Dm28c and to both hg38 and Dm28c, respectively.

### 2.5 miRNA alignment, annotation and quantification

Given the short length of mature miRNAs and that they often occur in families that share highly similar sequences, many reads in small RNA-seq datasets (around 46% in our case) have more than one best alignment which makes determining their true origins difficult. To deal with this multi-mapping problem, we used ShortStack (Axtell, 2013; Johnson et al., 2016), a tool that improves the placement of multi-mapping reads by an alignment strategy that uses local genomic context to guide decisions on proper placement of ambiguous reads. In particular, we run ShortStack in the -mmap f mode, where the placement of multi-mapping reads is guided by the density of all mapped reads in each 50 nt bin the read occurs in. The densities are calculated from all the unique reads and the multi-mapping reads, these contributing 1/n, being n the number of possible alignments. When the placement cannot be disambiguated, one of the alignment positions is chosen randomly, only if the read aligns to up to 20 different places (-ranmax 20). As ShortStack runs on bowtie, we kept the options -v1 and -m 50 and re-aligned the reads classified as hg38 and hg38-Dm28c. While we used all the reads to proper deal with multi-mapping reads, the final bam alignment files were filtered with samtools and custom scripts in order to keep only those aligned reads 18 to 28 nts in length, as this is the known length distribution for human and mammalian miRNAs (miRBase version 22; Bartel, 2018). Finally, reads were annotated as human miRNAs if they showed at least 75% same strand overlap with known human miRNAs in miRBase v22.1 (Griffiths-Jones et al., 2006; Kozomara et al., 2019). For this, BEDTools intersect v2.27.1 (Quinlan and Hall, 2010; Quinlan, 2014); -f 0.75 -s was used. In order to see if the parasite produces any small RNA which could mimic human miRNAs, those reads classified as Dm28c were also filtered to 18-28 nts and aligned with bowtie (same parameters as before) straight to the human miRNAs defined in miRBase.

### 2.6 Expression analysis of annotated miRNAs

We utilized the R package DESeq2 v1.36.0 (Anders and Huber, 2010; Love et al., 2014) to normalize and correct the data for the different sequencing depth of the libraries and to test for differential expression, based on the obtained read count matrix for human miRNAs. DESeq2 provides methods for differential analysis of count data by the use of negative binomial generalized linear models; additionally, DESeq2 incorporates shrinkage estimation for dispersions and fold changes to improve the stability and interpretability of estimates. A minimum pre-filtering of the count matrix was done to keep only those miRNAs that have at least 10 reads in at least two samples. Principal component analyses (PCA) were performed with FactoMineR v2.4 (Lê et al., 2008) and factoextra v1.0.7, based on transformed data by regularized logarithm (rlog, blind=TRUE) (Love et al., 2014) after selection of the top leading miRNAs (*i.e.* miRNAs with the highest sample variance). These were selected using the projection score (Fontes and Soneson, 2011) implemented following Breschi et al. (Breschi et al., 2016). Further clustering of samples used dendextend v1.16.0 (Galili, 2015). After the initial quality analysis, we separated the dataset into two. The “cell type dataset”, included only the samples taken at 24 hpi, in order to study the behaviour of the different cell types. The “cardiomyocyte dataset”, included all cardiomyocyte samples, intended to examine the response of this cell type at different post-interaction times.

To study differentially expressed miRNAs in the cell type dataset, a design formula with two factors, cell type and condition, was implemented. To determine which miRNAs showed a condition-specific effect while accounting for variability between cell types, we calculated a likelihood ratio test (LRT) with a reduced model which took into account only the cell type factor. To estimate the log2 fold changes (log2FC) between infection and control conditions, Wald tests were performed for both hypothesis |log2FC|>0 and |log2FC|>1. Shrinkage of log2FC estimates to control for small sample sizes and low read counts (*i.e.* obtention of shrunk log2FCs) was done by the apeglm method (Zhu et al., 2018) implemented in DESeq2. For multiple testing correction, we used a stratified false discovery approach, taking a per-condition Benjamini and Hochberg FDR-corrected p-value<0.01. miRNA expression levels between control and infected samples were also analyzed independently for each cell type with one variable design formulas (~condition). Wald tests, log2FC shrinkage and multiple testing correction were repeated as above. Expression heatmaps were based on rlog transformed data (blind=FALSE) and produced with the R pheatmap v1.0.12 package. Volcano plots were produced with the R/Bioconductor package EnhancedVolcano v1.14.0 (Blighe, 2018).

A first round of quality controls, including PCA, was performed to study the cardiomyocyte dataset. Then, samples collected at 0 and 3 hpi were re-classified together as “early” (or “0 + 3” hpi samples), to account for four replicates per condition at early infection time point. Differential expression analysis was performed for infected versus control samples at 0 + 3 hpi. For the complete cardiomyocyte dataset, we implemented a design formula with two factors, time and condition. To determine which miRNAs showed a condition-specific effect while accounting for the time factor, a LRT with a reduced model (~time) was performed. Wald tests, apeglm shrinkage and plots were performed as above.

### 2.7 Functional analysis of miRNAs

Functional enrichment of miRNA lists was performed in miEAA (Backes et al., 2016; Kern et al., 2020) and TAM 2.0 (Lu et al., 2010; Li J. et al., 2018). The over-representation analysis (ORA) was done using selected miRNAs in the context of all detected miRNAs in the specific cell type (the reference set). For ORA, all available categories were used, with p-value correction using FDR adjustment, for each category independently. Unless otherwise indicated, significant categories were identified for adjusted p-values <0.05. The miRNA-gene networks were created using miRnet (Fan et al., 2016; Chang et al., 2020), starting with miRNA or gene lists. In the case of miRNA lists, miRTarBase was used as the miRNA-target repository. To simplify the obtained networks, the shortest path or the minimum network options were selected. Network clustering was performed with the random walk-based InfoMap algorithm. A few related gene lists were explored using STRING (Szklarczyk et al., 2019; Szklarczyk et al., 2021); network clustering was performed with the Markov clustering algorithm (MCL, inflation parameter 3).

## 3 Results

### 3.1 Infection and miRNA profiles in the dual transcriptomes

Cardiomyocytes, HeLa and THP1-derived macrophages were infected with *T. cruzi* trypomastigotes, and miRNA response was evaluated at 24 hpi, and also at 0 and 3 hpi in the case of cardiomyocytes (**Figures S1A, B**). Cell type-specific infection rates were comparable, and infection index (Aoki et al., 2020) did not present significant differences (**Figures S2A, B**). Remarkably, trypomastigotes were able to widely infect all studied cell types.

**Figure 1:**
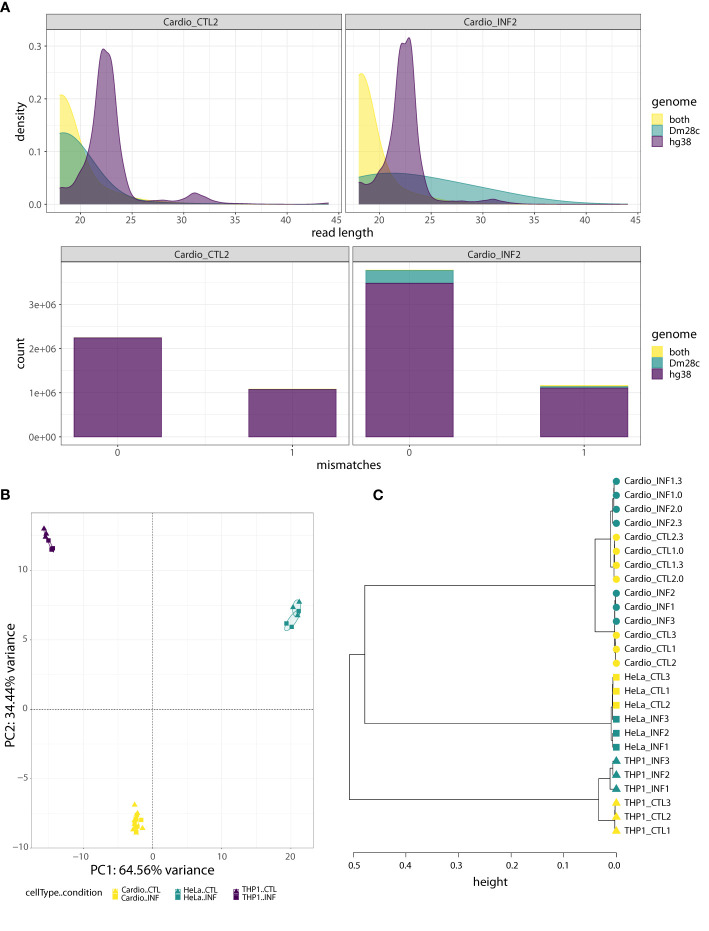
Human-cell response to *T. cruzi* Dm28c infection. **(A)** Quality analysis for one control sample (Cardio_CTL2) and one infected sample (Cardio_INF2), where reads were aligned to the joint hg38-Dm28c genome and classified as “hg38”, “Dm28c”, or “both” (“hg38-Dm28c”). Upper: the density plots show the read length distribution of aligned reads. The density peak for hg38 reads matches the expected length for miRNAs. On the contrary, most of the reads mapping in both genomes are very short, as are the reads assigned to Dm28c in the uninfected sample. In the infected sample, the Dm28c reads have a wide range of lengths rather than a dominant length. Bottom: the stacked barplots show the absolute frequency of “hg38”, “Dm28c” and “both” reads, considering the number of mismatches (0 or 1) in the joint genome alignment. Under both conditions, the fraction of hg38 reads is the highest, with the proportion of “both” class reads being almost irrelevant. With respect to reads categorized as Dm28c, they represent a minimal artifact in the uninfected sample, but have a non-negligible contribution in the infected sample. **(B)** PCA of miRNA expression levels, performed on the read counts of the 15 miRNAs showing the highest sample variance. Read counts were normalized and transformed by regularized logarithm (rlog). **(C)** Hierarchical clustering of control and infected samples (0, 3 and 24 hpi) based on sample-to-sample distances obtained from normalized and transformed expression counts of 641 miRNAs with at least 10 reads in at least two samples. The Ward’s linkage method of clusterization was applied on sample distances calculated as *1-corr*, being *corr* the Pearson correlation based on rlog values. Circles, squares and triangles represent cardiomyocytes, HeLa and THP1 samples, respectively. Yellow and green indicate control and *T. cruzi* infected samples, respectively.

Regarding the small RNA-seq data, after trimming and filtering steps, we aligned the reads to the joint “hg38-Dm28c” reference genome, and classified them into three partitions: those mapping only to the human genome (“hg38” reads), those mapping only to the *T. cruzi* genome (“Dm28c” reads) and those mapping to both human and parasite genomes (“hg38-Dm28c” reads; **Figure S1B** and **Table S1**). We also confirmed that the length distribution of hg38 reads showed an enrichment corresponding to the length expected for mammalian miRNAs (about 22 bases; **Figures 1A**; **S3A, B**). Interestingly, while reads aligning to the parasite genome (Dm28c and hg38-Dm28c reads) were short in uninfected samples, they increased in size and abundance after infection. For instance, reads classified as Dm28c were below 0.06% in the control samples but between 1 to 18% in the infected samples, having cardiomyocytes and THP1 above 5% of parasite reads (**Figures 1A**, **S3A, B**). This confirms our capacity to detect parasite-derived small RNAs in infected cells with confidence.

As expected based on our library preparation method, an average of 64% of hg38 reads were miRNAs, while they accounted for less than 4% in the hg38-Dm28c partition (and corresponded to miR-1260a, miR-1260b, miR-34c-5p, miR-151a-3p, miR-452-5p and miR-1246; **Table S1**). Small variations in miRNA length and sequence (isomiRs) were responsible for some reads corresponding to those miRNAs splitting between the hg38 and the hg38-Dm28c category. Addition or removal of hg38-Dm28c reads had negligible effects in host miRNA profiles (**Figure S4A**), although a few outliers could be observed (**Figure S4B**). Except for miR-1246, levels of these miRNAs in hg38-Dm28c were not modulated by infection (**Figures S4C, D**), arguing against their parasitic origin. Furthermore, miR-1246 was modulated by infection in both categories (hg38 and hg38-Dm28c) suggesting again that ambiguous reads actually come from the host. **Figures S5** and **S6** show detailed information about genome partition, length distribution and alignment mismatches for those reads annotated as miR-34c-5p and miR-1246 (using cardiomyocytes as a working example). Notably, all ambiguous reads align with one mismatch to *T. cruzi* reference, and we reason that this also suggests cross-mapping issues with sequences originated by host cell transcriptional activity. Finally, when we searched whether reads in the Dm28c partition could be highly similar to known human miRNAs, almost no matches were found in concordance with our bioinformatics strategy (**Table S1**).

### 3.2 A *T. cruzi*-host miRNA signature emerges above cell-type specificity

Considering the miRNA profiles built with the hg38 partition results, between 217 and 495 annotated miRNAs were detected per sample at a minimum depth of 10 reads (**Table S1**). Noticeably, five to ten explained 75% of the expression profile in each sample, with miR-21-5p always being the most expressed miRNA (**Figure S7**). A PCA of the 15 top leading miRNAs shows a clear separation among the cardiomyocytes, HeLa and THP1 samples (**Figure 1B**). A hierarchical clustering of the sample-to-sample distances based on the regularized transformed expression values of all expressed miRNAs complement and reinforce these results, and further differentiation according to condition and collection time is suggested (**Figure 1C**). Not surprisingly, cell identity is the main factor driving miRNA expression data (Ludwig et al., 2016; Patil et al., 2022).

**Figure 2:**
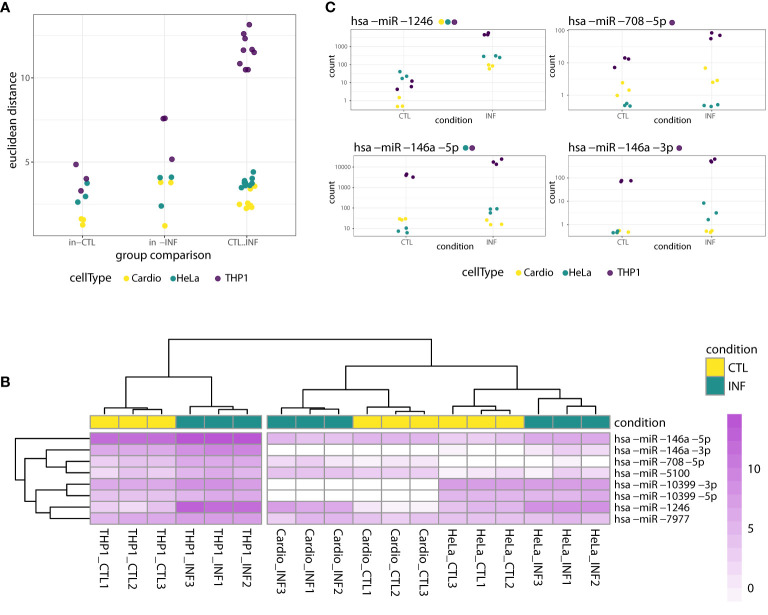
miRNA response to *T. cruzi* 24* hpi* infection considering both cell type and condition factors. **(A)** Dots represent sample-to-sample euclidean distances, based on normalized expression of all detected miRNAs, categorized as within-control, within-infection and between control-infection comparisons. For each cell type, there are three and nine within-group and between-group possible sample comparisons, respectively. **(B)** Heatmap of rlog expression values for the selected eight miRNAs with significant LRT (FDR-adjusted p<0.01) when ~cell type + condition *versus* ~cell type was compared. Values are scaled by row. Samples and miRNAs are clustered on euclidean distances. **(C)** Normalized gene counts of miR-1246, miR-708-5p, miR-146a-5p and miR-146a-3p in control and infected samples, which exhibited a significant LRT (FDR-adjusted p<0.01). Expression values are split by condition and colored by cell type. Although the LRT suggests a significant increase in expression upon infection for these miRNAs, not all showed a significant increase when analyzed separately for each cell type. Each miRNA name is accompanied by a colored point indicating if the miRNA was significantly increased in the corresponding cell type-specific Wald test (FDR-adjusted pvalue<0.01).

To identify miRNAs with altered expression in the different cell types, we conducted more in-depth analyses in the “cell type dataset”, that is, using only the samples obtained at 24 hpi for cardiomyocytes, HeLa and THP1. First, we estimated sample-to-sample distances and categorized them as within-control, within-infection and between control-infection groups. THP1 has the largest distances between control and infected samples (**Figure 2A**) so we can foresee a large effect of this cell type on statistical analysis not disaggregated by cell type. Next, we compared infected and control miRNA profiles with a generalized linear model that also included a factor for the cell type. For 26 miRNAs, the DESeq2-calculated LRT indicated that the condition plus cell type design formula better explains the data than a model including only a cell type factor (FDR-adjusted p-value<0.01, **Table S2**). However, when we considered the global log2FC between all infected and control samples, only eight miRNAs (miR-146a-5p, miR-146a-3p, miR-1246, miR-708-5p, miR-7977, miR-10399-5p, miR-10399-3p and miR-5100), corresponding to six different precursors, showed shrunk log2FC above 1. We consider that these miRNAs have a condition-specific effect, beyond the variability between cell types. In concordance, in a clusterization based on the expression of these miRNAs, samples segregate primarily according to cell type and secondarily according to condition, despite the fact that most of these miRNAs showed rather small within-cell type variations (**Figure 2B**). When we consider what is known about these miRNAs, miR-1246, miR-5100, miR-7977 and miR-10399 are rated as “not enough data” by miRBase (Kozomara et al., 2019), and not included in the curated miRNA database MirGeneDB (Fromm et al., 2015; Fromm et al., 2020). On the contrary, miR-146a is a well known miRNA with functions in inflammatory and immune processes (Kroesen et al., 2015), while evidence supporting an association between miR-708 and cancer has been accumulating (Monteleone and Lutz, 2017). Interestingly, both members of the mir-146 family (miR-146a and miR-146b), have been recently described as deregulated during *T. cruzi* infection, in *in vitro* (Farani et al., 2022), *ex vivo* (Medina et al., 2020) and *in vivo* experimental models (Navarro et al., 2015; Laugier et al., 2020; Ballinas-Verdugo et al., 2021). Regarding their cell-specific expression, and in agreement with our data, miR-146 is mainly expressed in immunocompetent cells, while miR-708 is near ubiquitous (although not expressed, for instance, in HeLa cells) (Patil et al., 2022). **Figure 2C** shows that miR-1246 expression increases dramatically upon infection in all three cell types, miR-708 increases in cardiomyocytes and THP1 but it is negligible in HeLa, and both miR-146a matures (5p and 3p) are upregulated upon infection in THP1 and HeLa. Most of these trends in the expression patterns were statistically significant in the cell type-specific Wald tests, as reported in the results below (and **Figure 2C** itself). Overall, while the main factor driving miRNA expression is cell identity, some miRNAs, notably miR-1246, miR-146a and miR-708, are strongly responsive to *T. cruzi* infection.

### 3.3 miRNA response to *T. cruzi* infection in non-immune cells

To better assess the response of each cell type to *T. cruzi* infection, we performed differential expression analysis with a generalized linear model with a one factor design (infected versus control) per cell type (24 hpi). In cardiomyocytes, while a hierarchical clustering of the sample-to-sample distances shows a separation between control and infected samples, only miR-1246 was considered upregulated based on established criteria (Wald test, |log2FC|>1, FDR-adjusted p<0.01) (**Figure S8A**; **Table S3**). None of the miRNAs mentioned in the literature associated with heart disease and/or Chagas disease showed any remarkable change at 24 hpi. miRNA response in cardiomyocytes is further discussed below.

**Figure 3:**
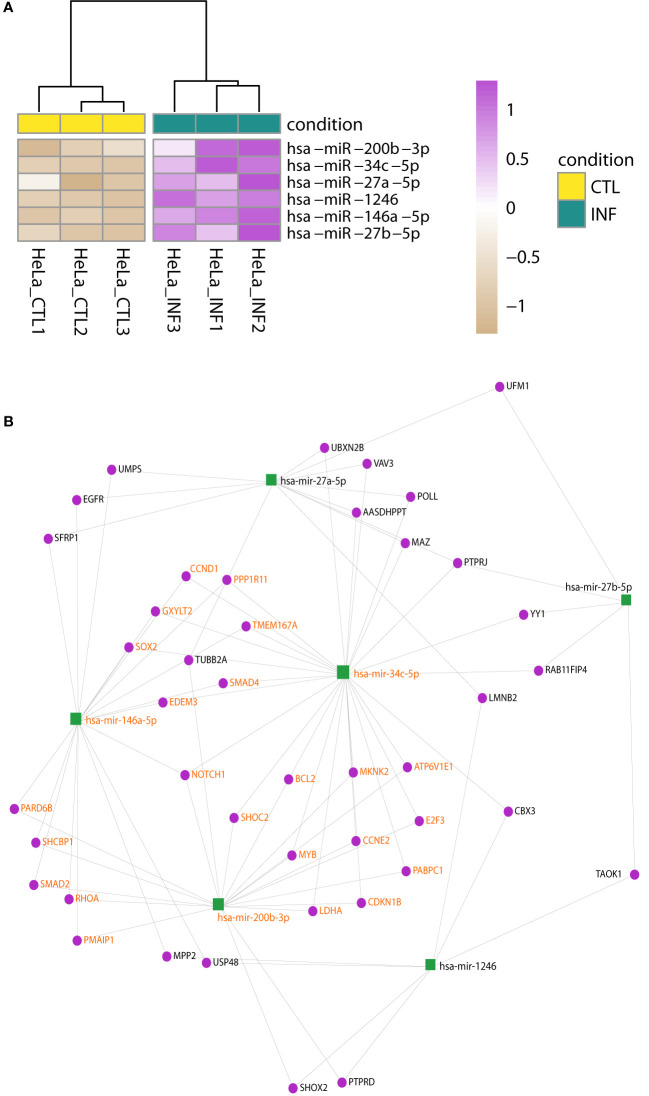
Changes in miRNA expression upon infection of HeLa cells. **(A)** Heatmap of rlog expression values of six miRNAs with differential expression upon *T. cruzi* infection of HeLa cells (Wald test, FDR-adjusted pvalue<0.01). Values are scaled by row. Samples are clustered on euclidean distances. **(B)** miRNA-gene interaction network built upon the list of the six upregulated miRNAs during *T. cruzi* infection of HeLa cells. A significant network module (InfoMap pvalue<0.001), which includes miR-34c-5p, miR-146a-5p and miR-200b-3p, was further identified. The functional enrichment of terms associated with the entire network is mostly explained by this module. Nodes are colored in green and purple for miRNA and genes, respectively. miRNA and gene labels in bold orange indicate the module members.

In HeLa, the hierarchical clustering again shows the split between control and infected samples (**Figure S8B**) and four miRNAs were detected with significant changes: miR-1246, miR-146a-5p, miR-200b-3p, miR-34c-5p (**Figure 3A**; **Table S4**). Matures miR-27a-5p and miR-27b-5p may have meaningful changes as well (Wald test FDR-adjusted p-value<0.05 and shrunk log2FC>0.5). An enrichment analysis of these six miRNAs using miEAA (Backes et al., 2016; Kern et al., 2020), selected a few WikiPathways (as Wnt signaling pathway and pluripotency) and 261 disease terms of the MNDR database (FDR-adjusted p-value<0.05) (Ning et al., 2021). Most of them were due to disease annotations of miR-146a-5p, miR-200b-3p and miR-34c-5p, highlighting the involvement of these miRNAs in relevant biological processes (**Table S5**). It is known that *T. cruzi* infection drives HeLa metabolic and cell signaling reprograming at the transcriptome level (Chiribao et al., 2014), being Akt kinase critical for parasite replication (Caradonna et al., 2013). We implemented a network approach in miRNet (Fan et al., 2016; Chang et al., 2020) to explore previous data generated for the same infection model in our group (Chiribao et al., 2014; Libisch, 2020). Although data is from the pre-replicative phase (6 hpi), the miRNA-target gene network obtained from downregulated genes predicted miR-146a-5p, miR-27a-5p and miR-200b-3p as important interactors (node degree above 15; **Table S5**). In order to gain insights into the potential regulatory roles of the six upregulated miRNAs in HeLa, we constructed a miRNA-gene network using these miRNAs as input (**Figure 3B**; **Table S5**). Functional enrichment analysis of the network using Reactome terms revealed significant involvement in signal transduction, cell cycle modulation and PI3K/Akt regulation, being EGFR, RHOA, NOTCH1, CDKN1B and CCND1 some of the key genes to this functionality of the network (**Table S5**). Notably, we also identified a significant subnetwork module (InfoMap pvalue<0.001, **Figure 3B**), involving miR-34c-5p, miR-146a-5p and miR-200b-3p, as well as most of their target genes in the network. This module was found to explain most of the functionality associated with the entire network (**Table S5**). These findings suggest that these miRNAs and their target genes may play important roles in regulating the signaling pathways active during HeLa response to *T. cruzi* infection.

### 3.4 THP1-derived macrophages show a broad response to *T. cruzi* infection

**Figure 4:**
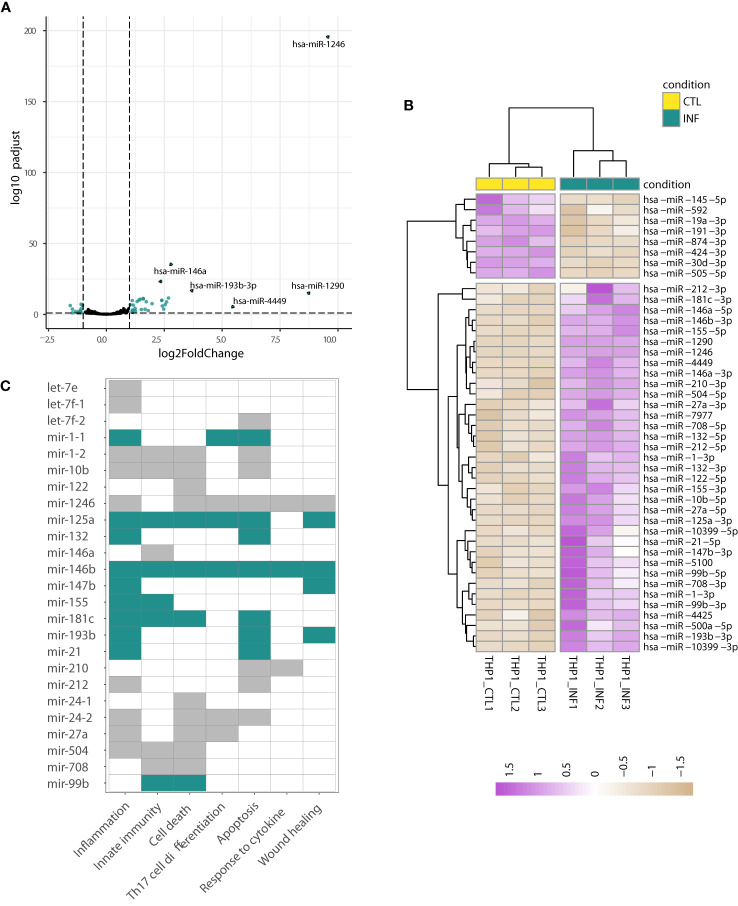
Changes in miRNA expression upon infection of THP1-derived macrophages. **(A)** Volcano plot shows the miRNA statistical significance (on the y-axis) over the log2 fold change after apeglm shrinkage (on the x-axis). Each dot represents a gene. Green dots correspond to a subset of 43 miRNAs differentially expressed (selected as those with Wald test FDR-adjusted p<0.01, apeglm shrinkage values |log2FC|>0.9 and mean of normalized counts of all samples >10). The top five upregulated miRNAs are labeled. **(B)** Heatmap of rlog expression values (blind=FALSE) for these 43 differentially expressed miRNAs after THP1 infection with *T. cruzi*. Values are scaled by row. Samples and miRNAs are clustered on euclidean distances. **(C)** Functional enrichment based on the upregulated miRNAs (performed in TAM). The GO:BP terms with FDR-adjusted p-value<0.05 are shown. The miRNAs contributing to each term are indicated by green and gray color (green is used when miRNA is a known immunomiR, gray otherwise). miRNA names are following TAM results.

When the above analyses were performed on the THP1-derived data, the hierarchical clustering of the sample-to-sample distances confirmed the split between THP1 infected and control samples (**Figures 1A**, **2A**; **S8C**; **Table S6**). Concordantly, 61 miRNAs showed differential expression after *T. cruzi* infection (Wald test, FDR-adjusted p-value<0.01) with 15 meaningfully upregulated when a specific |log2FC|>1 was considered. Other miRNAs of interest, such as miR-21-5p and miR-210-3p, also showed considerable changes (*e.g*. shrunk |log2FC|>0.9). miR-1246, miR-1290, miR-4449, miR-193b-3p and miR-146a were the top five upregulated miRNAs (**Figure 4A**). The heatmap in **Figure 4B** represents scaled expression values of the 43 most impacted miRNAs upon infection (those with shrunk |log2FC|>0.9 and FDR-adjusted p-value<0.01). A miRNA-gene network built from these 43 miRNAs as seeds shows let-7e-5p, let-7f-5p, miR-24-3p, miR-21-5p and miR-155-5p as hubs (node degree>50; **Figure S9**), reinforcing the potential important role of these miRNAs in the response of the THP1-derived macrophages to the parasite insult.

Several of these most upregulated miRNAs (*e.g*. miR-155, miR-146a, miR-146b, and miR-21) are considered “immunomiRs” (Kroesen et al., 2015; Chandan et al., 2020). They are widely expressed in cells of the immune system and regulate critical immune functions. Noteworthy, they are major regulators of Toll-like receptor (TLR) signaling; some miRNAs are induced by TLR activation whereas others target 3’ untranslated regions of mRNAs encoding components of the TLR signaling system (Taganov et al., 2006; O’Neill et al., 2011; Quinn and O’Neill, 2011). The functional analysis of these upregulated miRNAs carried out in TAM (Lu et al., 2010; Li J, et al., 2018) showed, concordantly, a significant enrichment in functions related to inflammation, innate immunity, cell death and apoptosis, among others (**Table S7**). As shown in **Figure 4C**, miR-155, miR-210, miR-21 and miR-146a, have an important role in driving the functional enrichment result. Two well-known targets of miR-21 and miR-146a, transforming growth factor beta-1 (TGFB1) and sprouty homolog 2 (SPRY2), were already found downregulated in THP1 cells during early infection (Libisch, 2020). Complementarily, we used Libisch et al. dataset (Libisch, 2020) to explore which mediators could be driving THP1 response to infection. We carried out a network analysis of the upregulated genes, followed by posterior gene clustering. We identified two top gene sets in the network, the first centered in TNF and NF-kB and the second enriched in interferon type I and type III signaling (**Table S7**, see discussion).

On the other hand, the miRNA-gene network based on the 18 downregulated miRNAs is less intricate, being miR-19a-3p and miR-19b-3p the most interconnected nodes (**Table S7**). The network showed functional enrichment in terms “regulation of apoptotic process”, “regulation of programmed cell death” and “regulation of cell cycle” of the gene ontology, biological process domain (GO:BP; FDR-adjusted p-value<0.001, **Table S7**). A complementary functional analysis performed in TAM confirms the involvement of ten miRNAs in cell death and cell cycle functions (**Table S7**). One of the enriched transcription factors, Fli-1 proto-oncogene (FLI1), which is involved in transcription of the let-7a miRNAs, was found downregulated in *T. cruzi*-infected THP1 cells at early and intermediate time points (Libisch, 2020). Conversely, suppressor of cytokine signaling 1 (SOCS1) and TNFAIP3 interacting protein 1 (TNIP1), known targets of miR-19a, miR-19b, miR-30e and miR-221 (Karagkouni et al., 2018; Huang et al., 2022), resulted with increased expression in the same dataset. SOCS1 is also a target of miR-155, which is upregulated in our data, illustrating the complexity of miRNA-mRNA interaction networks.

### 3.5 miRNA dynamics in *T. cruzi* infected cardiomyocytes

**Figure 5:**
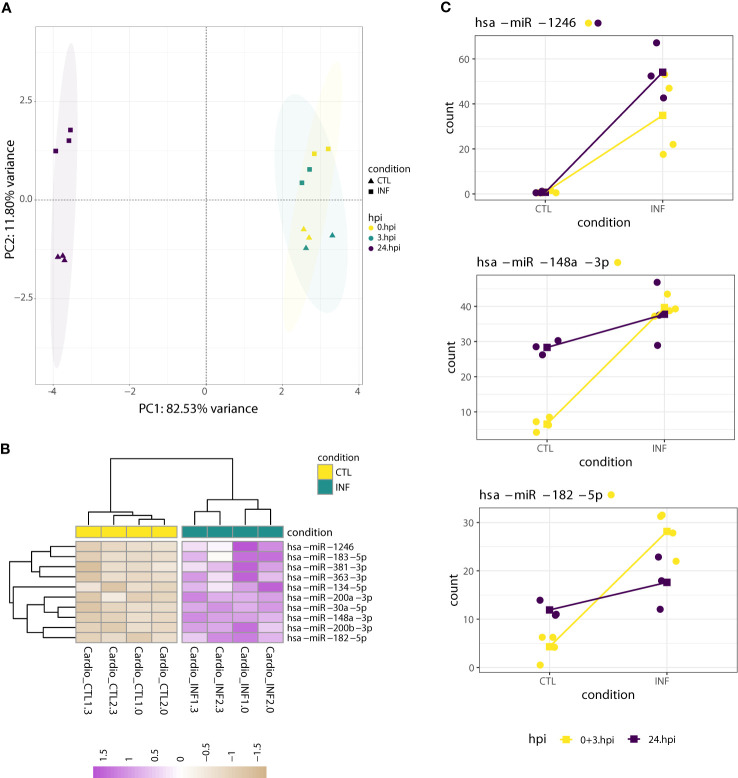
miRNA dynamics in cardiomyocytes at early (0 + 3 hpi) and 24 hpi time points. **(A)** PCA of miRNA expression levels, performed on the read counts of the nine miRNAs showing the largest variance. Read counts were normalized and transformed by rlog. As the PCA highlights, most of the variance can be explained by expression changes between samples taken at 0 or 3 hpi and those collected at 24 hpi. This allowed the re-classification of all 0 and 3 hpi samples to “early” ones (0 + 3 hpi). **(B)** Heatmap of rlog expression values (blind=FALSE) for ten differentially expressed miRNAs after cardiomyocyte infection with *T. cruzi* at early time post-interaction (Wald test FDR-adjusted p-value<0.01). Values are scaled by row. Samples and miRNAs are clustered on euclidean distances. **(C)** Normalized counts of miRNAs which exhibited a significant LRT (FDR-adjusted p<0.01) in *T. cruzi*-infected cardiomyocytes. miRNA expression values were grouped by condition and colored by early (0 + 3 hpi) or 24 hpi timepoint. Dots correspond to sample expression values while squares represent mean expression counts per condition and timepoint. Lines connect time-paired mean values across condition. Although the LRT suggests a significant increase in expression upon infection for these miRNAs, only miR-1246 showed a significant increase when analyzed separately in both timepoints. Each miRNA name is accompanied by a colored point indicating if the miRNA was significantly increased in the corresponding time-specific Wald test (FDR-adjusted pvalue<0.01).

We previously showed that infected cardiomyocytes present extensive transcriptomic changes at earlier time points of infection (Libisch et al., 2018). Other authors also described cardiomyocyte changes at the RNA -and also structural- level after 48 and 72 h post interaction (Pereira et al., 2005; Manque et al., 2011; Melo et al., 2019). The minimal number of miRNAs modulated by *T. cruzi* infection in cardiomyocytes at 24 hpi (*i.e.* only miR-1246 was deregulated) by no means have to be representative of what happens during the whole parasite cycle (*i.e.* from cell invasion to parasite release). We reasoned that, considering the 0 - 24 hpi timeline, miR-1246 might be the only responsive miRNA, or additional miRNAs could be transiently deregulated. To distinguish between these two possibilities, we also performed RNA-seq at 0 and 3 hpi. In this “cardiomyocyte dataset”, a PCA based on expression levels of the nine top leading miRNAs shows a clear separation between samples collected at 24 hpi from those obtained at 0 and 3 hpi (**Figure 5A**). As samples taken at 0 and 3 hpi were similar enough (see also **Figure 1C**), we re-classified them as “early” infection samples. In this setting with four and three biological replicates per condition at early (0 + 3 hpi) and amastigote replicative (24 hpi) phases, respectively, we proceeded with the differential expression analyses. Notably, whereas miR-1246 was the sole miRNA deregulated at 24 hpi, ten miRNAs, including miR-1246 itself, were upregulated in *T. cruzi* infected cardiomyocytes during the early phase of infection (Wald test, FDR-adjusted p-value<0.01; **Figure 5B**; **Table S8**). All these differentially expressed miRNAs kept log2FC above 1 after apeglm shrinkage, although low average expression values raise some caution. The results suggest a more important role for miRNA-mediated post-transcriptional regulation networks at early stages of parasite infection.

Known “myomiRs” (muscle/cardiac miRNAs) such as miR-1 and miR-133 (McCarthy, 2008) were barely detected in our experiment. We revised the estimations of their expression levels according to the GTEx project and, for instance, miR-1 is expressed very low in cardiomyocyte derived cells, being this in accordance with our results (GTEx Portal, n.d; Lonsdale et al., 2013). On the other hand, miRNAs upregulated during mice cardiomyocyte differentiation were observed, such as miR-18a, miR-23b, miR-26a, miR-182, miR-183 and miR-200a/b (**Table S8**) (Joladarashi et al., 2014). In fact, miR-182-5p, miR-183-5p, miR-200a-3p and miR-200b-3p are included in our upregulated list. Functional analysis of our differentially expressed miRNAs using miEAA showed that, among many disease terms, there is significative enrichment in cardiac-related terms, including “dilated cardiomyopathy” and “cardiomegaly” (**Table S9**). A direct consultation of NMRD, a non-coding RNA-disease association database (Cui et al., 2018; Ning et al., 2021), pointed that all ten upregulated miRNAs are related to cardiac disease terms with confidence score above 0.9. The miRNA-gene network constructed from the upregulated miRNAs indicates that miR-30a-5p, miR-200b-3p, miR-182-5p and miR-183-5p have the highest centrality in the network, adding support for their role in the regulation of targets involved in cardiomyocyte response to *T. cruzi* infection (**Table S9**; **Figure S10**). A functional analysis of the genes incorporated in the network highlights a role in the regulation of PI3K/Akt signaling pathway (**Table S9**), due to the presence of CREB1, CDKN1A, GSK3B, TNRC6A and NOTCH1 genes (**Figure S10**). Of note, these genes were not found downregulated in previous transcriptomic analysis of human and mouse cardiomyocytes infected with *T. cruzi* at early and 24 hpi (Manque et al., 2011; Udoko et al., 2016; Libisch et al., 2018; Libisch, 2020; Venturini et al., 2023). However, this is not surprising considering miRNAs mainly downregulate mRNAs at the translational level, not necessarily affecting mRNA abundance. Remarkably, miR-182, miR-183, miR-200a/b, miR-30a and miR-381-3p have already been associated to regulation of PI3K/Akt pathway, although in the context of carcinogenesis (Ghafouri-Fard et al., 2021). In summary, despite the absence of more traditional cardiac miRNAs, we found upregulation of miRNAs previously associated with both cardiac-related terms and PI3K/Akt regulation. It is worth mentioning that, during early infection, PI3K/Akt signaling triggers the elevation of cytosolic Ca^2+^ concentration, promoting the actin cytoskeleton disruption and lysosome mobilization required for effective parasite internalization (Ferri and Edreira, 2021).

Next, we explored the miRNA dynamics in *T. cruzi*-infected cardiomyocytes along early (0 and 3 hpi) and amastigote replicative phase (24 hpi) time points. We established in DESeq2 a generalized linear model that included both time and condition factors. A time effect comparison (24 hpi versus early) would involve samples from different processing batches and sequencing depths (**Table S1**) that cannot be properly corrected, so we did not analyze this. Instead, we compared the infection effect (infection versus control condition) and found three miRNAs showing a condition-specific effect. The LRT indicated that for miR-1246, miR-148a-3p and miR-182-5p, the condition plus time design formula better explained the data than a model including only the time factor (FDR-adjusted p-value<0.01, **Figure 5C**; **Table S10**). miR-363-3p also showed a significant LRT, but its average normalized expression is below 10 and was not further evaluated. Considering all results, we can establish a few observations. First, miR-1246 is the sole miRNA with significant upregulation along early and replicative phases of infection in cardiomyocytes. This is remarkable because miR-1246 is also the only miRNA consistently upregulated by infection at 24 hpi in all three cell lines tested in this study. Second, miR-148a-3p and miR-182-5p are also upregulated during the early phase of parasite infection, but this effect is lost at later time points. Still, they show a tendency to be more abundant in infected samples at 24 hpi (**Figure 5C**), which explains the significant LRT result. For the other seven miRNAs upregulated at early infection times, the LRT results and expression patterns reinforce that they have more transient changes being important at early infection times.

## 4 Discussion

In this work, we used our well-known host cell-*T. cruzi* infection models and small RNA sequencing to study the host transcriptional response at the miRNA level. Our small RNA-seq data has a dual nature and in fact, although *T. cruzi* lacks the canonical miRNA-mediated silencing machinery, some parasite small RNAs are packaged and released in extracellular vesicles with relevance in the host-parasite interaction (Garcia-Silva et al., 2010; Lye et al., 2010; Garcia-Silva et al., 2014). Thus, we combined different bioinformatics tools to identify, to the best of our abilities, the origin of each read (**Figure S1B**). As expected, most of the reads (>80%) were classified as hg38, while about 2% was of ambiguous origin. In infected samples, Dm28c reads were 1-18% (above 5% in cardiomyocytes and THP1) although they were not similar enough to any known human miRNA. In control samples, where reads with a parasite origin are not expected, we found about 0.06% Dm28c reads, that are exclusively explained by misalignments, despite we allowed at most 1 mismatch. These reads are shorter, reinforcing this issue. Whereas this 0.06% of Dm28c reads did not affect our expression profiles, they indicate that we have the same underlying phenomenon in the hg38-Dm28c partition. In fact, regarding the ambiguous reads, they are also shorter and only 4% of them were assigned to miRNAs (while this number was 64% for the hg38 partition). Needless to say, these short-alignment issues are something that cannot be overcome, at least completely, when we deal with small RNA-seq data. Many genes are similar members of the same RNA family, making matters worse. Other confounding factors are sequencing errors, quality of the used genomic references and biological true positive “mismatches” related to extensive post-transcriptional editing of the RNA species (Wyman et al., 2011; Correia de Sousa et al., 2019). In the particular case of miRNAs, specific algorithms to handle multiple alignments and cross-mapping given the observed mismatch pattern were proposed (Hoon et al., 2010), but their implementation was not successful here. In spite of this, the hg38-Dm28c ambiguous partition, with the sole exception of miR-1246 (see below), did not contribute significantly to our miRNA profiles either. Ambiguous reads annotated as human miRNAs were very few and were present in both control and infected samples, thus not contributing to our differentially expressed miRNAs. Of note, while the misalignment and cross-mapping issues of hg38-Dm28c reads do not quantitatively affect our miRNA profiles, this may not be true in the case of other small RNA species. Beyond bioinformatics, and as expected for a parasite that does not have miRNA-mediating silencing mechanisms, *T. cruzi* did not show any miRNA-like mimic of known human miRNAs capable of a role in the host-parasite interaction.

Regarding miR-1246, it is the most upregulated miRNA in our dataset and, undoubtedly, a common *T. cruzi* infection signature to all three host cell types. Some ambiguous reads mapped to this miRNA, but solely in the case of the infected samples, thus we cannot discard that, at least few of the reads, may have a true origin in the parasite transcriptional activity. However, given the high upregulation and increased number of reads in infected compared to control samples (**Figure 2C**), as well as the reduced length and the absence of reads mapping without mismatches to the Dm28c genome (**Figure S6**), a much more plausible explanation is that some of the reads originated by the host cell transcriptional activity end up misaligned to the parasite genome as well. No follow-up experiments were conducted; therefore, we cannot completely rule out the possibility of other sources of cross-contamination.

Short sequences are not only problematic due to their ambiguous genome-of-origin classification, but could affect small RNA annotation more broadly. For instance, miR-1246 itself would not be a miRNA, but would correspond to misassigned fragments derived from the U2 small nuclear RNA (RNU2) (Baraniskin et al., 2013; Mazières et al., 2013; Zhang et al., 2021; Tosar et al., 2022). Curated miRNA-target databases register its association with AGO2 (Karagkouni et al., 2018; Huang et al., 2022), although this does not imply effective silencing of targets (Gottwein et al., 2011). Despite there is not much information about its function, miR-1246 has been widely detected in studies that look for cancer biomarkers (Takeshita et al., 2013; Zhang et al., 2016) and it has been attributed a role in oncogenesis (Chen et al., 2014; Sun et al., 2014; Zhang et al., 2016). Notably, Kanlikilicer et al. recently demonstrated that exosomal miR-1246 released in abundance from ovarian cancer cells confers chemo-resistance via targeting the Cav1/p-gp/M2-type macrophage axis (Kanlikilicer et al., 2018). All in all, this miR-1246/RNU2-derived fragment deserves more study in general and, in particular, in the context of *T. cruzi* infection. We are currently undergoing further research on this.

Clearly, THP1 infected cells have a response that differs sharply from that of cardiomyocytes and HeLa. This work involves bulk small RNA sequencing so it is important to consider whether quantitative differences among the host cell responses may be explained by different percentages of trypanosome-infected cells. As we noted above, THP1 cells are no more extensively infected (**Figure S2**) so this does not appear to be an underlying explanation for their increased response. Our results indicate that these professional phagocytic cells, compared to cardiomyocytes or HeLa cells, are capable of a stronger response at the miRNA level to *T. cruzi* infection, in concordance with previous studies where dendritic cells or macrophages were challenged with intracellular pathogens (Furci et al., 2013; Siddle et al., 2015). This is also consistent with the response of these cell types to *T. cruzi* challenge at the mRNA level (Libisch, 2020). It is established that internalization of trypomastigotes in macrophages involves TLR2 interaction with the parasite surface, activation of PI3K pathway signaling and Rab5 activation for phagosome formation (Maganto-Garcia et al., 2008), which makes unsurprising the observation of extended changes in RNA species. In addition, previous works have reported increased miRNA basal expression of macrophages (Graff et al., 2012) and this may contribute with additional statistical power to detect significant changes when RNA-seq is used. Finally, given the impact of a higher amount of pathogen recognition receptors, intracellular mediators and the inflammatory signals released by themselves, it would be interesting to evaluate host-*T. cruzi* response separately in directly infected versus macrophages exposed to inflammatory stimuli, as it was carried out with other kinetoplastid models (Beattie et al., 2013).

Macrophage infection with kinetoplastids has been previously studied at the miRNA level, but these studies were conducted mainly in *Leishmania* (reviewed in Rojas-Pirela et al., 2022). A comparison of our 61 deregulated miRNAs showed only four coincidences (miR-24, miR-210, miR-221 and miR-425) to a set of 21 miRNAs altered when human primary macrophages were infected with *L. major* for 24 hours (Lemaire et al., 2013). Other works also revealed little convergence in the results (Geraci et al., 2015; Singh et al., 2016). The different models and, in particular, the different technologies used to quantify miRNA expression, prevent further analysis of these differences (e.g. Lemaire et al. used TaqMan human microRNA arrays). In another study, THP1 monocytes infected with *L. major*, resulted in 48 deregulated miRNAs (Nimsarkar et al., 2020). In this case, 13 miRNAs were coincident with our study. Furthermore, the authors defined a signature of nine miRNAs (miR-146a-3p, miR-146a-5p, miR-122, miR-155-3p, miR-155-5p, miR-188, miR-9-3p, miR-9-5p, and miR-147) found to play a key role in the inflammatory response to *Leishmania*, that widely overlaps with our results.

miRNAs have emerged as pivotal regulators of macrophage polarization and understanding their participation and functions in regulating this process is important for discussing disease progression and developing novel miRNA-based therapeutic strategies (Wu et al., 2016; Li H, et al., 2018; Curtale et al., 2019). Graff et al. studied the miRNAs involved in the different macrophage activation phenotypes (Graff et al., 2012). While our data is consistent with M1 classical pro-inflammatory phenotype, the upregulation of miR-193b and miR-27a suggests a mixture with M2 alternative activation phenotypes (driven by inductors as IL-4 and Fcɣ receptor stimulation) (Graff et al., 2012). Furthermore, concomitant upregulation of both miR-146a and miR-146b also points to a system buffering the expression of pro-inflammatory genes (Curtale et al., 2019). This is concordant with a previous study that suggested that infection by kinetoplastids results in a macrophage activation that is more similar to M2 and deactivation than to an M1 phenotype (Zhang et al., 2010). The authors showed that mouse macrophages infected with *T. cruzi* and *Leishmania* at 24 hpi, clustered together with macrophages stimulated by IL-17, IL-10 and IL-4, although signatures of interferon response were also present. However, we revised previous data of our group (Libisch, 2020) and it may suggest the participation of TNF, NF-kB and interferon type I and III mediators. These discrepancies with Zhang et al. can be attributed to the used models (*e.g.* human versus mouse, Dm28c versus CAI-72 *T. cruzi* strain), and to the different stage in the infection cycle (early infection in the case of the mRNA data we used).

miR-210, one of the upregulated miRNAs upon THP1 infection, has an important role in cancer (Dang and Myers, 2015) and recently was described as an “inflammomiR” (Virga et al., 2021), showing induced expression in response to invading pathogens, including *T. brucei*. Thus, miR-210 enters the immunomiRs list. While there is a clear role of miR-210 in modulation of host-pathogen interaction that is dependent on pathogen presence (Lemaire et al., 2013; Kumar et al., 2018; Virga et al., 2021), the particular parasite and host molecules involved in deregulation of miR-210 by *T. cruzi* are yet to be elucidated. Overall, in THP1 we found that differentially expressed miRNAs, specially those upregulated, may be involved in the shape of macrophage activation, perhaps toward a mixed M1-M2 phenotype, which could aid parasite survival.

According to its medical relevance, miRNA changes associated to CCC have been extensively studied. The findings have been recently reviewed and lists of miRNAs associated to chagasic cardiomyopathy presented (Acuña et al., 2020; Paul et al., 2020; Rojas-Pirela et al., 2022). Both myomiRs (miR-1, miR-133 and miR-208) and immunomiRs (miR-21, miR-155, miR-146a/b, miR-142), together with other miRNAs such as miR-29b, miR-30a, miR-138, miR-149 and miR-200b, have been found deregulated upon *T. cruzi* infection. In particular, miR-21, has been identified as a potential target for the treatment of CCC and other cardiac diseases (Bang et al., 2014; de Lucia et al., 2017; Nonaka et al., 2021). The original studies involved the analysis of acute and chronic *T. cruzi* infection, in mice models of CD (Navarro et al., 2015; Ferreira et al., 2017; Nonaka et al., 2019; Ballinas-Verdugo et al., 2021; Farani et al., 2023) and/or human patients, using either heart and/or serum samples (Ferreira et al., 2014; Linhares-Lacerda et al., 2018; Nonaka et al., 2019; Laugier et al., 2020; Nonaka et al., 2021). Fewer studies assessed miRNA changes at the level of cardiac cells (Monteiro et al., 2015). From all these miRNAs, only miR-30a-5p and miR-200b-3p are included in our list of upregulated miRNAs. While the lack of deregulation in myomiRs may come as a surprise, the discrepancy observed in immunomiRs can be attributed to differences in the experimental models used (Libisch et al., 2021). Our study utilized human cardiomyocytes, whereas most of the cited studies employed mouse cells. It is important to note that our samples were collected at 0, 3 and 24 hpi, and thus did not capture the complete dynamics of miRNA expression, including later time points when *T. cruzi* infection is known to induce extensive RNA and structural changes in cardiac cells (Pereira et al., 2005; Manque et al., 2011; Melo et al., 2019). Notably, a recent study using *T. cruzi*-infected rat cardiomyoblasts did not detect changes in the expression levels of miR-145-5p and miR-146b-5p until 48 hpi (Farani et al., 2022). Furthermore, comparing miRNA expression changes in cardiac cell cultures to those in heart tissue samples may result in even greater discrepancies. Cardiomyocytes are about 90% of heart mass but only 30% of the cells, and together with fibroblast and resident macrophages, they constitute a “three-in-a-box” cellular set that orchestrates cardiac processes (Psarras et al., 2019). It is established that the inflammation and fibrosis associated with *T. cruzi* infection and many other insults is related to the activity of the fibroblasts (Coelho et al., 2018), macrophages and infiltrating immune cells. In particular, CCC is characterized by monocyte and T-cell rich myocarditis with cardiomyocyte damage, hypertrophy and prominent fibrosis, whereas *T. cruzi* parasites are very scarce (Laugier et al., 2020). Therefore, miRNA expression changes measured by bulk transcriptomics, which averages the expression changes in all present cells of the heart sample, may not be representative of what is occurring in the cardiomyocytes. In this sense, *in vitro* studies of cardiac cells are relevant to help us understand their precise role in the CD development. Our work contributes to the understanding of whether cellular processes relevant to CD pathogenesis or treatment are potentially regulated at the post-transcriptional level by miRNAs. This information is valuable for identifying potential targets for intervention.

Our findings suggest that miRNA changes may be more prominent at the early stages of *T. cruzi* infection (0 and 3 hpi) compared to the amastigote replicative phase (24 hpi). These collection times entailed a 2 h period of interaction between trypomastigotes and cardiomyocytes. This time allows the activation of host signaling pathways due to parasite presence which may produce host-changes through free mediators or mediators released via extracellular vesicles, attachment to host cells and invasion itself, a plethora of events enough to differentiate early samples from control ones. Accordingly, there is evidence that extracellular amastigotes are capable of selectively triggering complex signaling pathways (by activation of Akt and ERK molecules) in HeLa cells using 5 to 90 mins incubation times (Ferreira et al., 2019). Regarding the regulation of miRNA expression itself, it is complex and turnover depends on both biogenesis and degradation rates. Biogenesis involves the transcription of pri-miRNA and several maturation steps (Ha and Kim, 2014; Trieber et al., 2019), while degradation involves enzymatic activities that can modify miRNA ends by adding or removing nucleotides or through a target-dependent mechanism. As it is understood, miRNA degradation rates can change quickly (Marzi et al., 2016) and this can have an impact on miRNA expression levels, even in the absence of relevant changes due to biosynthesis. miRNAs with “fast” decay rates can give rise to sharp and quick changes in miRNA levels (Marzi et al., 2016), potentially leading to the observed differences in miRNA expression at 0 and 3 hpi. However, overall, and in agreement with the study by Farani et al. (2022), our work suggests that miRNA-mediated post-transcriptional regulation may not play an extended role in gene expression changes in cardiomyocyte response to *T. cruzi* during the studied stage of infection (0 to 24 hpi).

**Figure 6:**
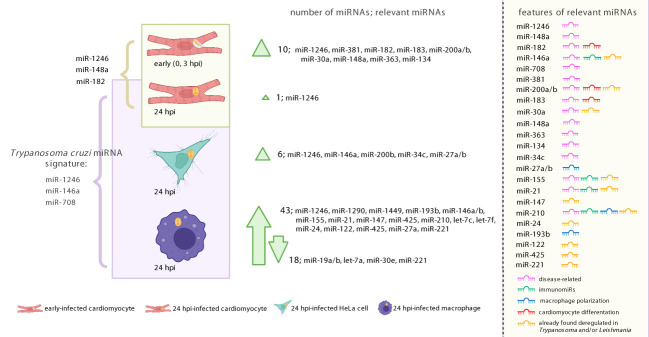
Graphical illustration summarizing our results. To the left, the figure shows most miRNAs identified as regulated upon *T. cruzi* infection (both Wald and LRT tests results). To the right, the list of relevant miRNAs indicates whether they have been associated with disease, immune response, macrophage activation, cardiomyocyte differentiation. It is also indicated if the miRNA was previously reported as regulated during *Trypanosma* and/or *Leishmania* infection. Created with BioRender.com.

We established that parasite infection is associated by moderate miRNA changes in cardiomyocytes (mainly at the early time point) and HeLa, while, as expected and discussed, professional macrophages showed a broad response. We detected mostly upregulated miRNAs upon infection. Few of them, noticeably immunomiRs miR-146a/b, miR-155 and miR-21, had been previously described as deregulated during *T. cruzi* infection and even in CD patients. We explored the putative functional implications of deregulated miRNAs. Not surprisingly, differentially expressed miRNAs were related to cell-type specific functions, as those related to phagocytosis or myocardium in THP1 and cardiomyocytes, respectively. **Figure 6** summarizes our results. Despite the main factor driving miRNA expression is cell identity, we were able to find a miRNA signature in the response of our studied cells to *T. cruzi* infection, during the amastigote replicative phase (24 hpi). The common signature involves the upregulation of miR-1246, miR-146a and miR-708 and deserves further exploration. miR-146a is a well-known immunomiR and already recognized as a recurrent deregulated miRNA during *T. cruzi* infection (Acuña et al., 2020; Paul et al., 2020; Rojas-Pirela et al., 2022). However, its expression is more or less restricted to immunocompetent cells. While our results regarding miR-146a cannot be extrapolated to all cell types, it seems to be a crucial player in many of the cells relevant for this trypanosmatid infection and CD. On the contrary, miR-708 is widely expressed, and it has been linked to several diseases, including cancer and neurodegeneration (Dileepan et al., 2014; Monteleone and Lutz, 2017). In this sense, it remains to be established if it could have a role in *T. cruzi* infection of other relevant cell types in CD, as fibroblasts and adipocytes, which express miR-708 but lack miR-146a (Lonsdale et al., 2013). Finally, to the best of our knowledge, this is the first study that reveals the importance of miR-1246 during *T. cruzi* infection, which could be related to our bioinformatics design. miR-1246 appears highly upregulated in our dataset, in a cell-type- and time-independent manner. Somewhat amusingly, this cell-type-unspecificity of miR-1246 is consistent with it not being a bona fide miRNA. Morevoer, as miRNA-1246/RNU2-derived fragments can be expressed in any cell type, our study opens an opportunity in the CD field to explore new biomarkers and therapeutic targets beyond miRNAs.

We acknowledged the limitations of our work related to ambiguous alignments and cross-mappings when analyzing small RNAseq data. Another limitation was the low number of biological replicates for early *T. cruzi* infection in cardiomyocytes (only two replicates per condition at 0 and 3 hpi). To address this, and enabled by their similarity, we analyzed combined samples from 0 and 3 hpi. Additionally, we refrained from explicitly analyzing the temporal effect of infection by comparing samples from 24 versus 0 + 3 hpi, as they were obtained from separate experiments. It is worth noting that our RNA-seq data represents an average of both infected and non-infected cells, which may introduce false negative detection of DE miRNAs. It would be beneficial for future studies to investigate parasitized cells separately from the neighboring non-infected ones, as Beattie et al. did (Beattie et al., 2013). The not-so-recent development of single-cell RNA-seq paves the way for taking this factor into account. HeLa and THP1 cells were used as models of epithelial cells and macrophages, respectively. Although these are transformed cell lines and some results may not be reproducible in normal cells, they have been widely and successfully employed in *T. cruzi* research. Lastly, it is important to note that our work is based on bioinformatics analysis of small RNA-seq data, and no follow-up experiments were conducted. Therefore, our conclusions represent new hypotheses that require further testing.

## Data availability statement

The raw sequence data were deposited in NCBI with accession number BioProject PRJNA607998 (https://www.ncbi.nlm.nih.gov/bioproject/PRJNA607998).

## Author contributions

NR performed all the bioinformatics data analysis and wrote the manuscript. ML performed all the experiments. CRob conceived and designed the experiments. CRov contributed with data analysis design and discussion of preliminary results. ML, JT and CRob commented extensively on the manuscript. All authors contributed to the article and approved the submitted version.
