# Supplementary material for: Comparative microRNA profiling of Trypanosoma cruzi infected human cells
Source: Front Cell Infect Microbiol. 2023 Jun 21;13:1187375. doi: 10.3389/fcimb.2023.1187375 (PMC10322668; doi:10.3389/fcimb.2023.1187375)
Supplement: Supplementary file 1 [file DataSheet_1.pdf]

Supplementary Material

Comparative microRNA profiling of *Trypanosoma cruzi* infected human cells

Natalia Rego, María Gabriela Libisch, Carlos Rovira, Juan Pablo Tosar, Carlos Robello*

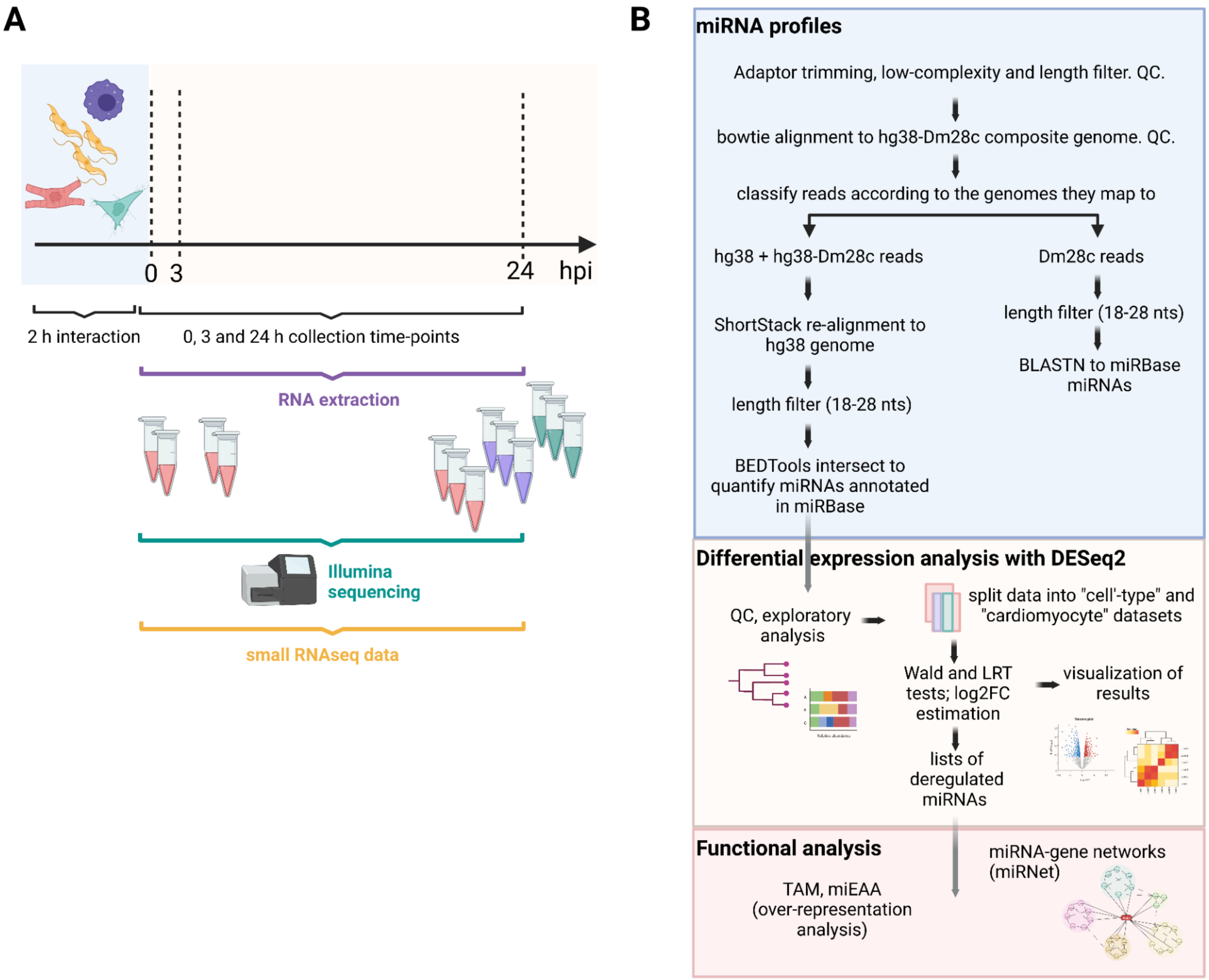

Figure S1. Experimental and bioinformatics design. **(A)** Experimental strategy used. *T. cruzi* Dm28c strain parasites (yellow) were incubated for 2 h with cardiomyocytes (red), HeLa (turquoise) or THP1-derived macrophages (violet). Samples were collected at 24 h post-interaction for all three cell

types. Additional cardiomyocyte samples were taken at 0 and 3 hpi. Non-infected control samples are not shown. **(B)** small RNA sequencing data analysis workflow. Created with BioRender.com.

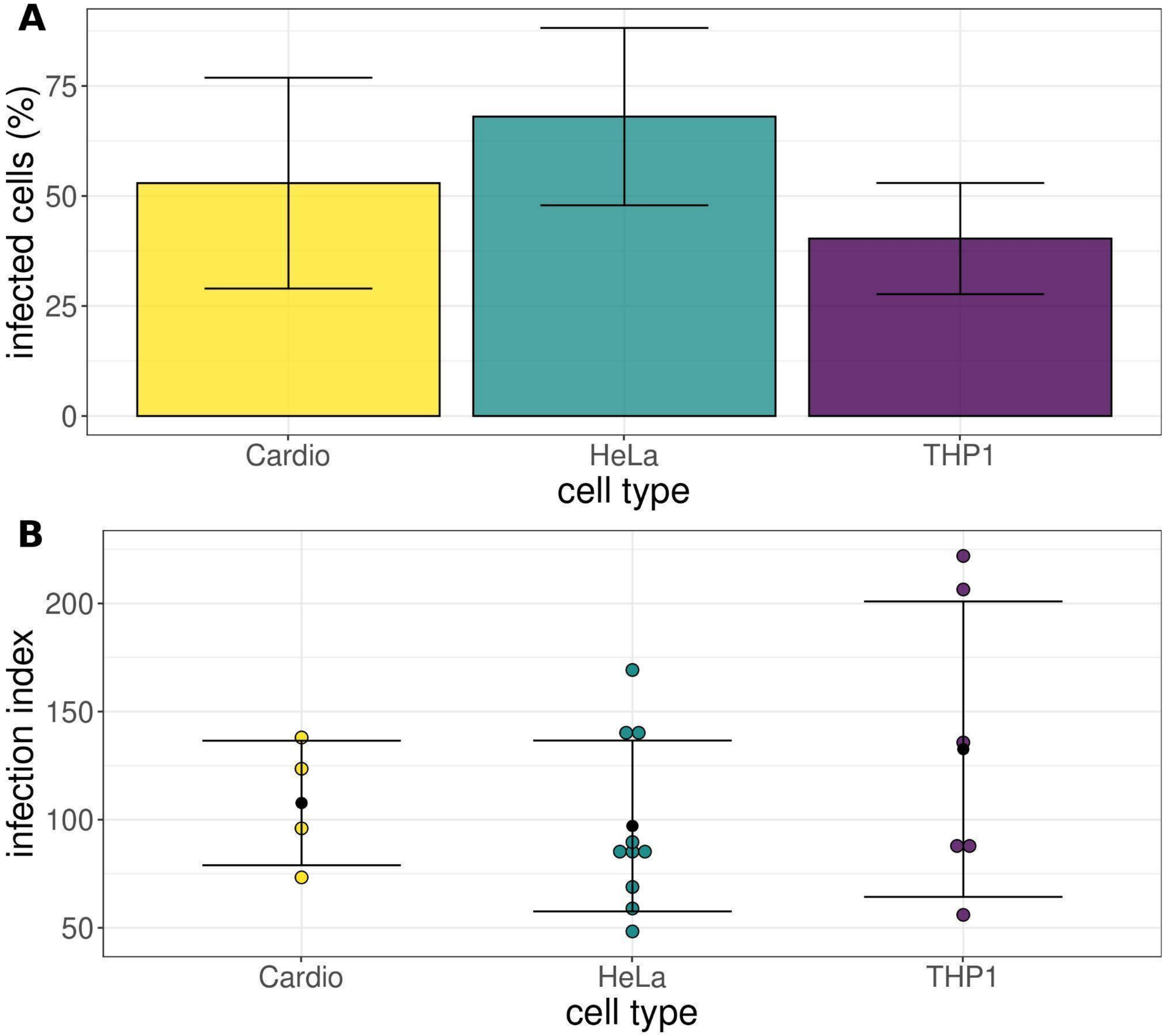

Figure S2. Human cell response to *T. cruzi* infection. **(A)** Barplots indicate the average percentage of infected cells at 24 hpi, for cardiomyocytes, HeLa and THP1 cells. Error bars represent the standard deviation. Estimations were obtained after counting infected and total cells using fluorescent microscopy on DAPI-dyed samples. The average percentage of infected cells was different between cell types, being 52.9 ± 24.0%, 68.0 ± 20.1% and 40.3 ± 12.6% for cardiomyocytes, HeLa and THP1, respectively (Chi-squared test; pvalue<0.01). **(B)** Dotplots show infection index values estimated for the three studied cell types at 72 hpi. Mean and standard deviation are also shown (black). As the number of amastigotes at 24 hpi is very low, particularly in THP1 cultures, infection index values

were estimated in 72 hpi infection assays. THP1 presented the highest infection index, although dispersion was also large (mean ± sd was 107.7 ± 28.8, 97.1 ± 39.5 and 132.6 ± 68.3 for cardiomyocytes, HeLa and THP1, respectively) and no significant differences were found (Kruskal-Wallis test; pvalue>0.5).

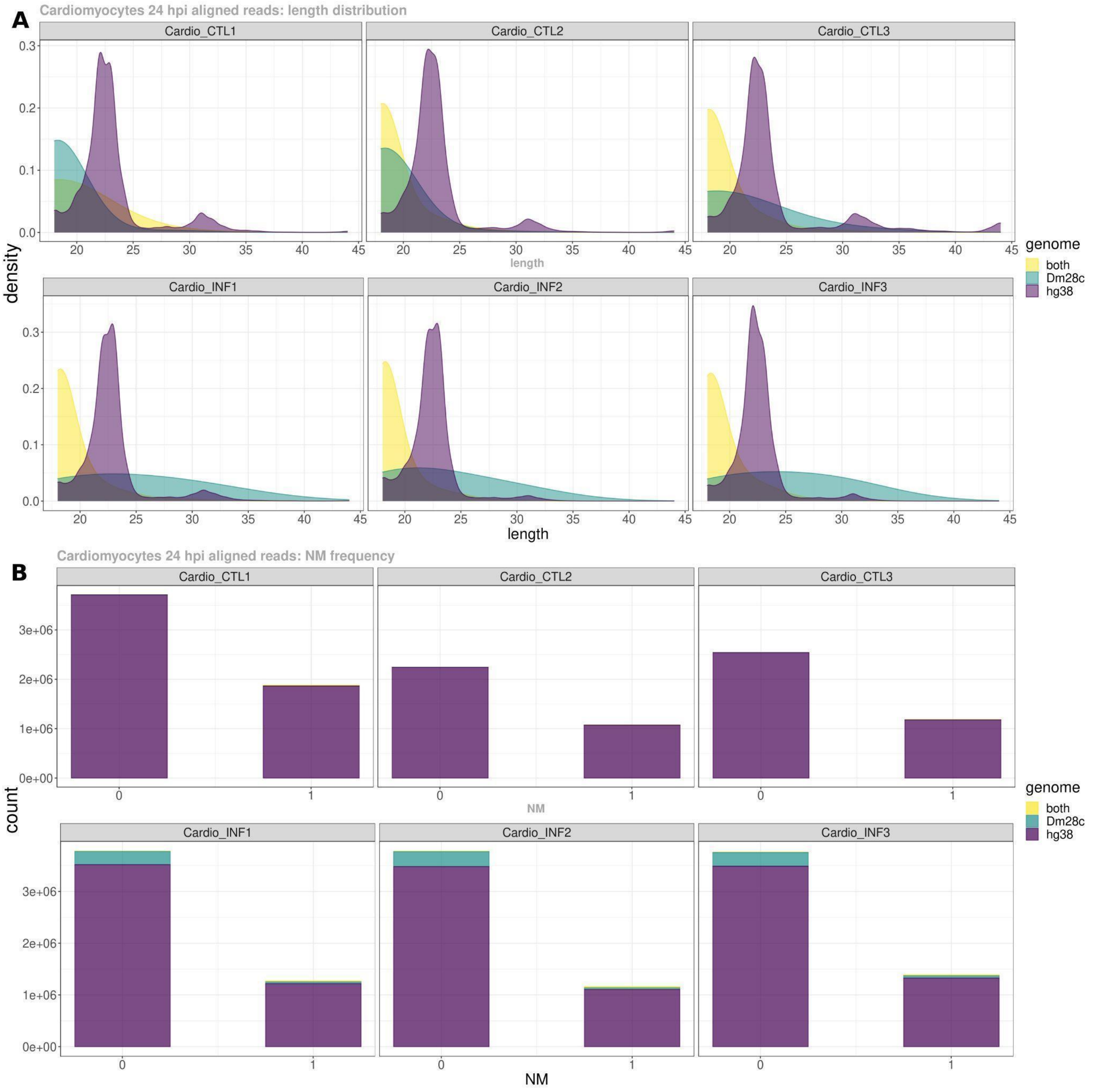

Figure S3. Quality control of aligned reads, taken cardiomyocytes at 24 hpi as an example (control and infected samples). **(A)** Read length distribution of aligned reads to the hg38-Dm28c composite genome. The density plot shows a clear peak around 22 nt in the case of the six samples when the "hg38" partition is considered. **(B)** For reads aligning with 0 and 1 mismatch to the joint genome (NM=0 and NM=1, respectively), the barplots show the number of "hg38", "Dm28c" and "both hg38 and Dm28c" genome partitions, for the non-infected and infected cardiomyocyte samples. Remarkably, there is an increase of reads assigned to the Dm28c partition in the *T. cruzi* infected samples but they do not show any sharp peak in their length distribution.

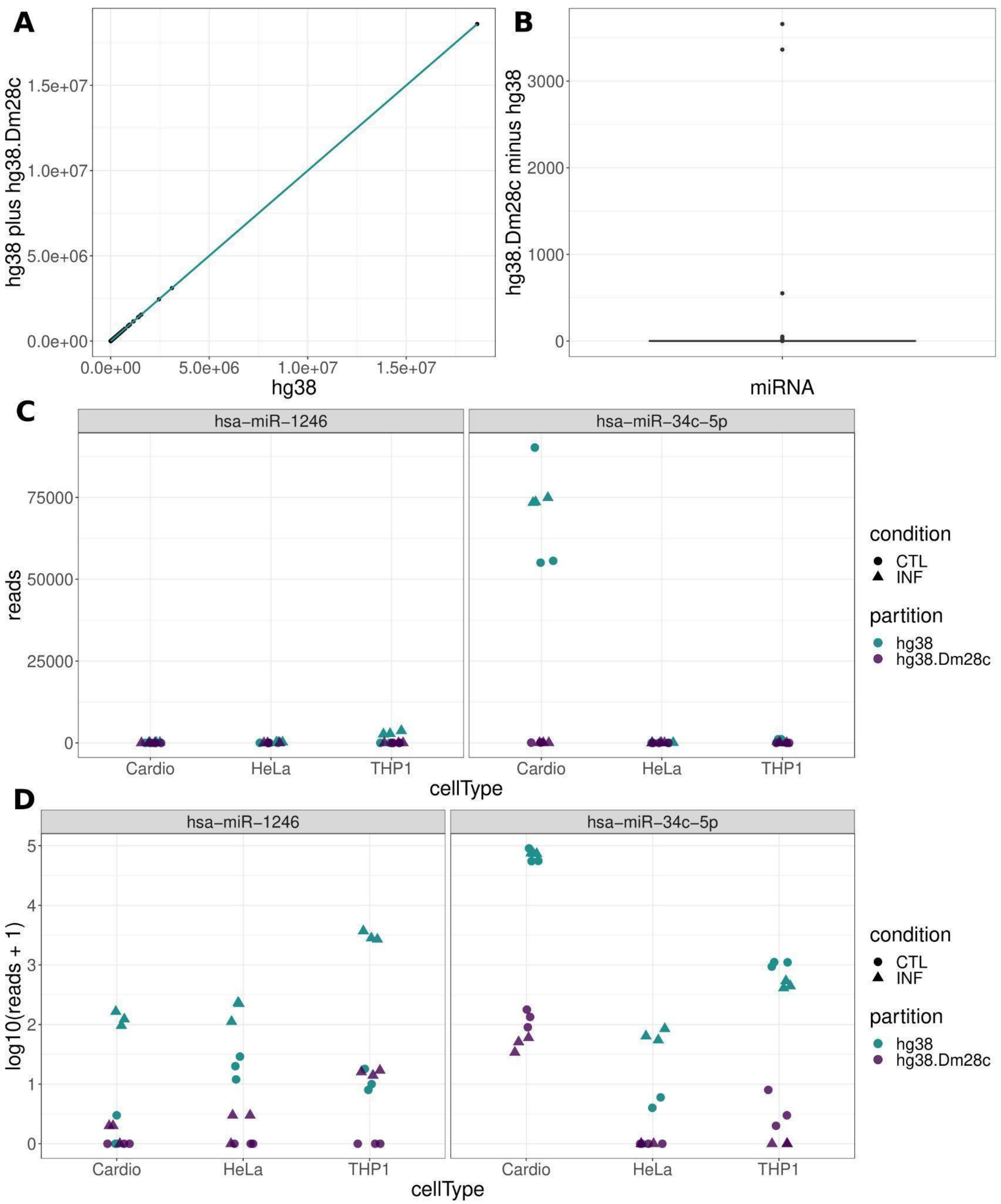

Figure S4. Contribution of the reads of the hg38-Dm28c partition to the miRNA expression profiles. **(A)** For each detected miRNA in the whole dataset, the plot represents the total raw counts considering the hg38 reads alone or from hg38 plus hg38-Dm28c partitions, being both values fairly

similar. **(B)** Boxplot showing the contribution of the hg38-Dm28c reads to the total raw count. For most miRNAs, the contribution is zero. Few of them contribute with more than 15 reads to the miRNA estimated read count (the outliers in the boxplot). **(C)** For miRNAs hsa-miR-1246 and hsa-miR-34c-5p the contribution is not negligible. In the case of miR-34c, reads aligned to both genomes and assigned to hsa-miR-34c-5p can also be seen in control samples. Because the parasite is absent, this demonstrates the existence of misalignment issues. On the other hand, in the case of miR-1246, the hg38-Dm28c reads assigned to this miRNA are only found in the infected samples. However, it is highly probable that this involves misalignment issues too, which is reinforced by the fact that miR-1246 expression also increases with infection when only the hg38 reads are considered. Certainly, we cannot discard a truly contribution from small RNAs with a parasite origin and in any case this deserves further study. **(D)** Same as (C) but using log10 transformation of raw counts in order to improve visualization.

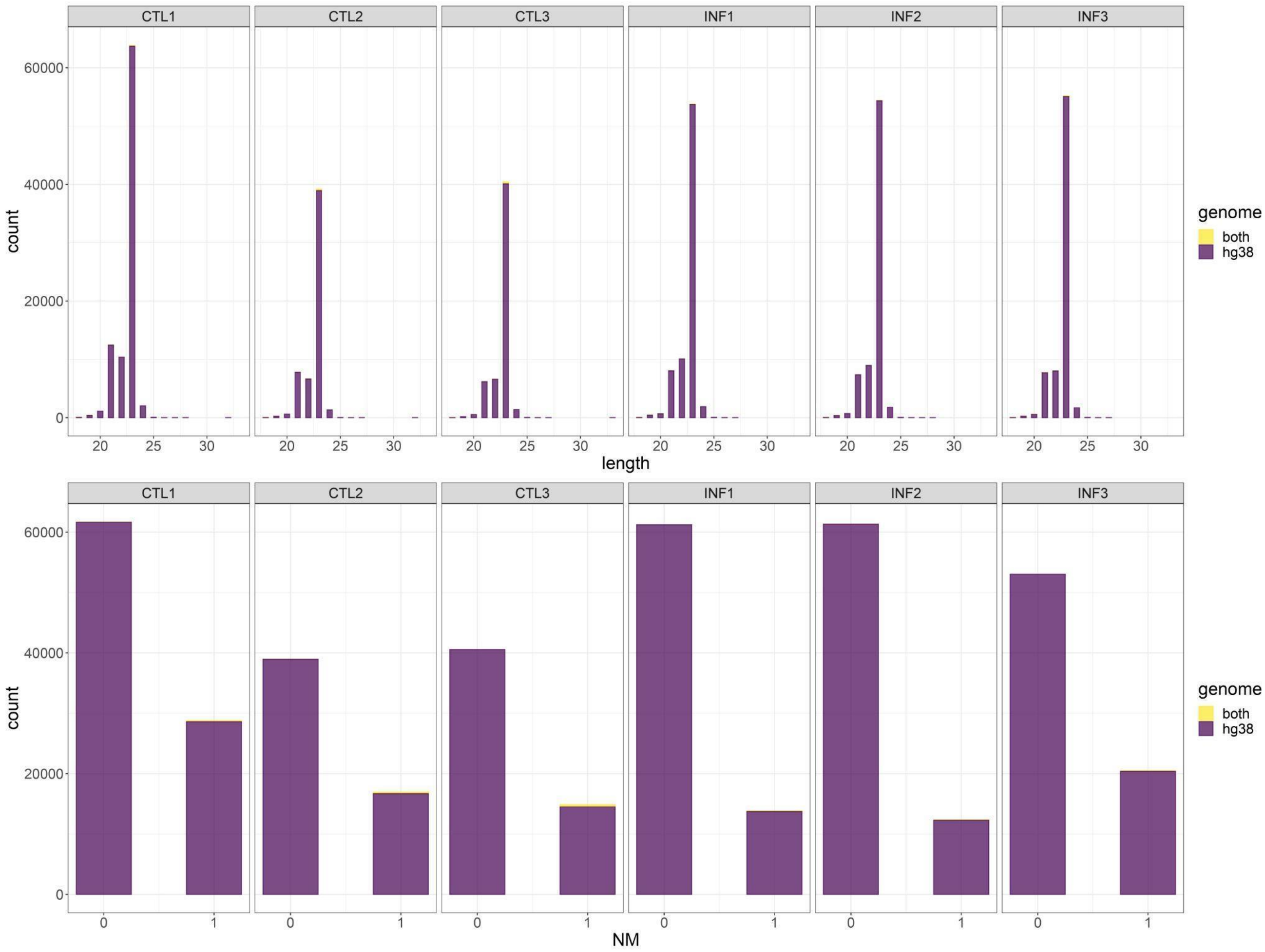

Figure S5. Features of reads annotated as hsa-miR-34c-5p, taken cardiomyocytes at 24 hpi as an example (control and infected samples). **(A)** Read length distribution of aligned reads to the hg38-Dm28c composite genome. The barplot shows a clear peak around 23 nt and, most importantly, ambiguous reads are quantitatively irrelevant and also present in the control samples (see samples CTL2 and CTL3) where the parasite was not present. **(B)** For reads aligning with 0 and 1 mismatch to the joint genome (NM=0 and NM=1, respectively), the barplots show the number of "hg38", "Dm28c" and "both hg38 and Dm28c" genome partitions for the non-infected and infected cardiomyocyte samples. Remarkably, those reads mapping to both genomes have one mismatch to the references; ambiguous reads annotated as miR-34c-5p but identical to the Dm28c genome were not identified.

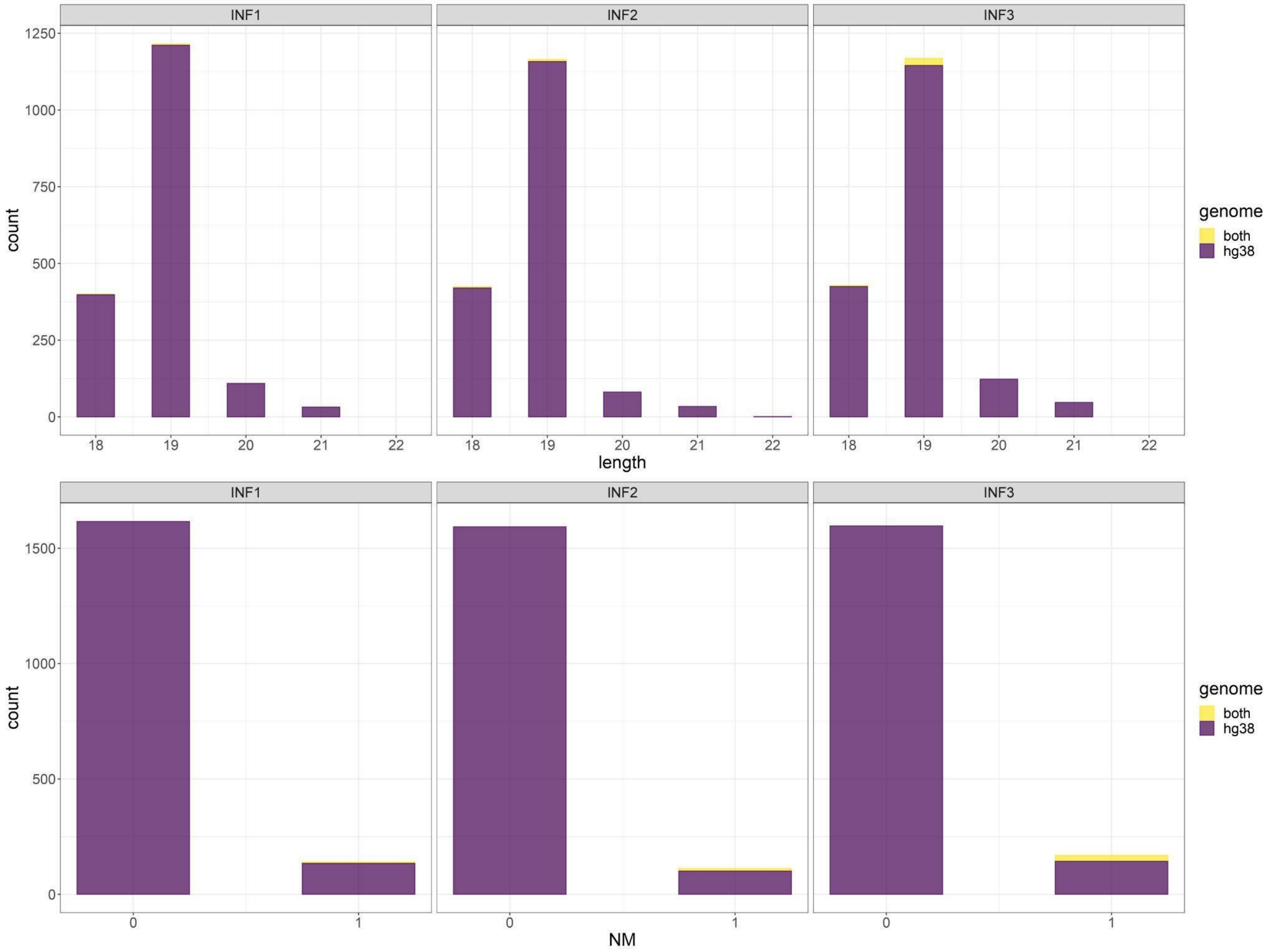

Figure S6. Features of reads annotated as hsa-miR-1246, taken cardiomyocytes at 24 hpi as an example. Since only zero, one, two, or six reads annotated as miR-1246 were identified in the control samples, we present only the data from the infected samples. **(A)** Read length distribution of aligned reads to the hg38-Dm28c composite genome. The barplot shows a clear peak around 19 nt, implying that miR-1246 is shorter than canonical miRNAs. Most importantly, ambiguous reads do not contribute in a relevant way to total read counts. **(B)** For reads aligning with 0 and 1 mismatch to the joint genome (NM=0 and NM=1, respectively), the barplots show the number of “hg38”, “Dm28c” and “both hg38 and Dm28c” genome partitions. Remarkably, those reads mapping to both genomes have one mismatch to the references; ambiguous reads annotated as miR-1246 but identical to the Dm28c genome were not identified.

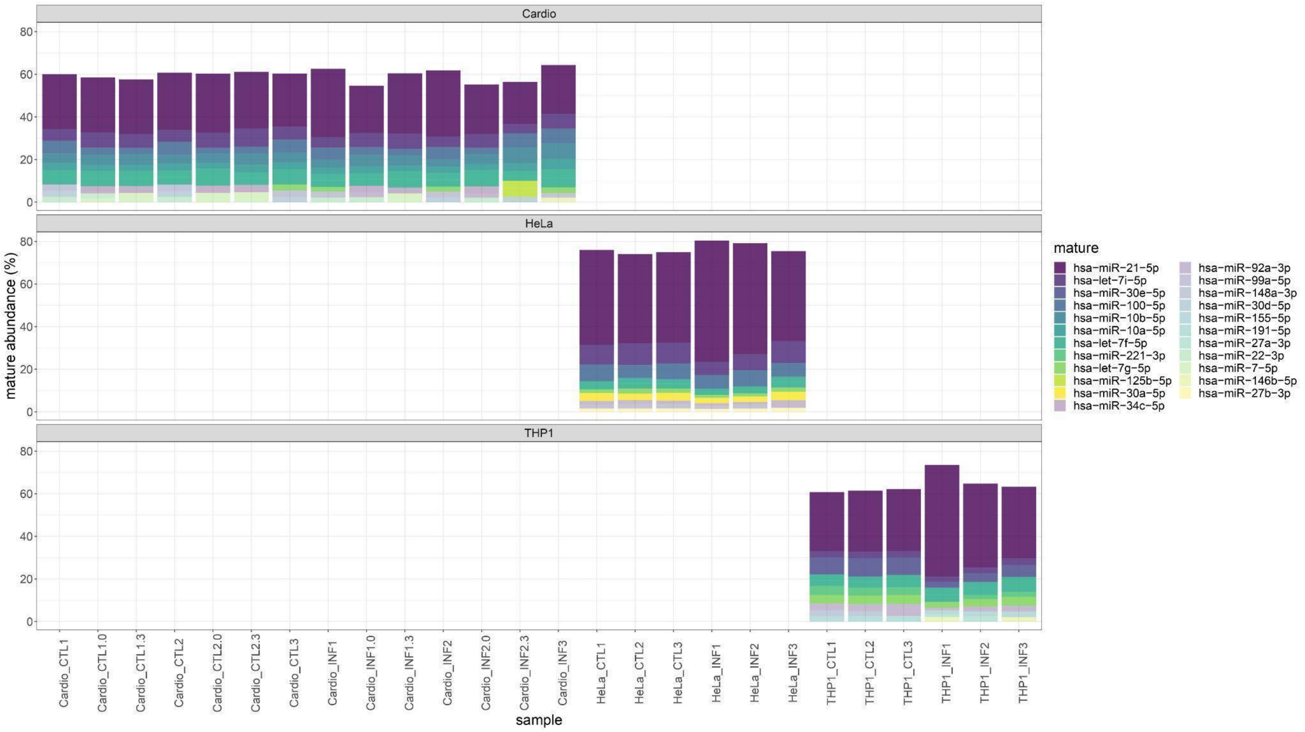

Figure S7. Top 10 expressed miRNAs per sample. The relative expression (DESeq2 normalized values) of the most expressed miRNAs per sample is shown. Samples from differentes cell types are shown in different facets of the plot. hsa-miR-21-5p is always the most expressed miRNA. Five to ten miRNAs per sample account for 75% of the sequenced reads annotated as miRNAs.

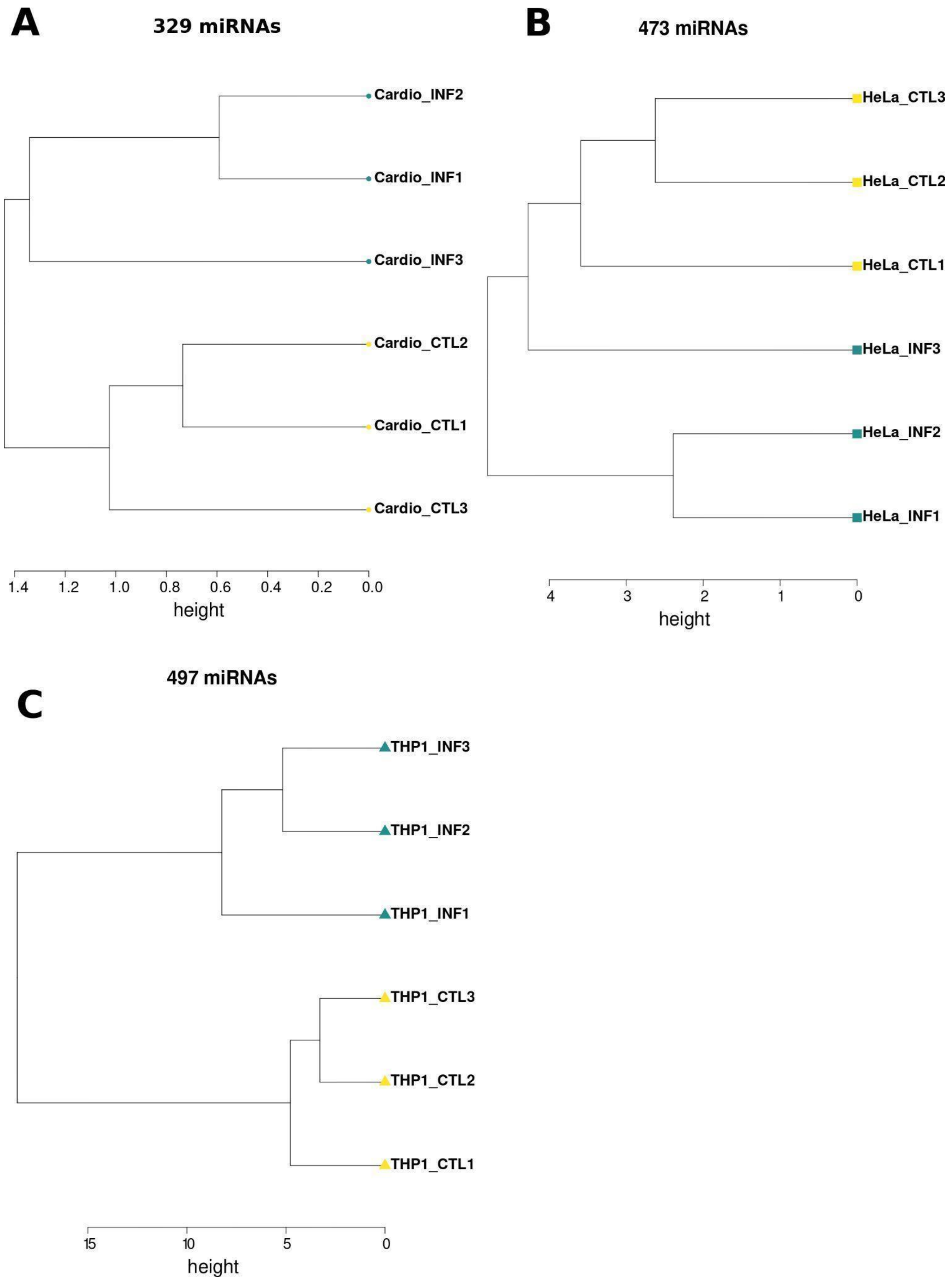
A
329 miRNAs
Cardio_INF2
Cardio_INF1
Cardio_INF3
Cardio_CTL2
Cardio_CTL1
Cardio_CTL3
1.4
1.2
1.0
0.8
0.6
0.4
0.2
0.0
height
B
473 miRNAs
HeLa_CTL3
HeLa_CTL2
HeLa_CTL1
HeLa_INF3
HeLa_INF2
HeLa_INF1
4
3
2
1
0
height
C
497 miRNAs
THP1_INF3
THP1_INF2
THP1_INF1
THP1_CTL3
THP1_CTL2
THP1_CTL1
15
10
5
0
height

Figure S8. Hierarchical clustering of control and infected samples (24 hpi) based on sample-to-sample distances obtained from normalized and transformed expression counts of miRNAs with at least 10 reads in at least two samples. The Ward's linkage method of clusterization was applied on sample distances calculated as *1-corr*, being *corr* the Pearson correlation based on rlog values. **(A)** Hierarchical clustering of cardiomyocyte samples based on expression values of 329 miRNAs. **(B)** Hierarchical clustering of HeLa samples based on expression values of 473 miRNAs. **(C)** Hierarchical clustering of THP1 samples based on expression values of 497 miRNAs. Circles, squares and triangles represent cardiomyocytes, HeLa and THP1 samples, respectively. Yellow and green indicate control and *T. cruzi* infected samples, respectively.

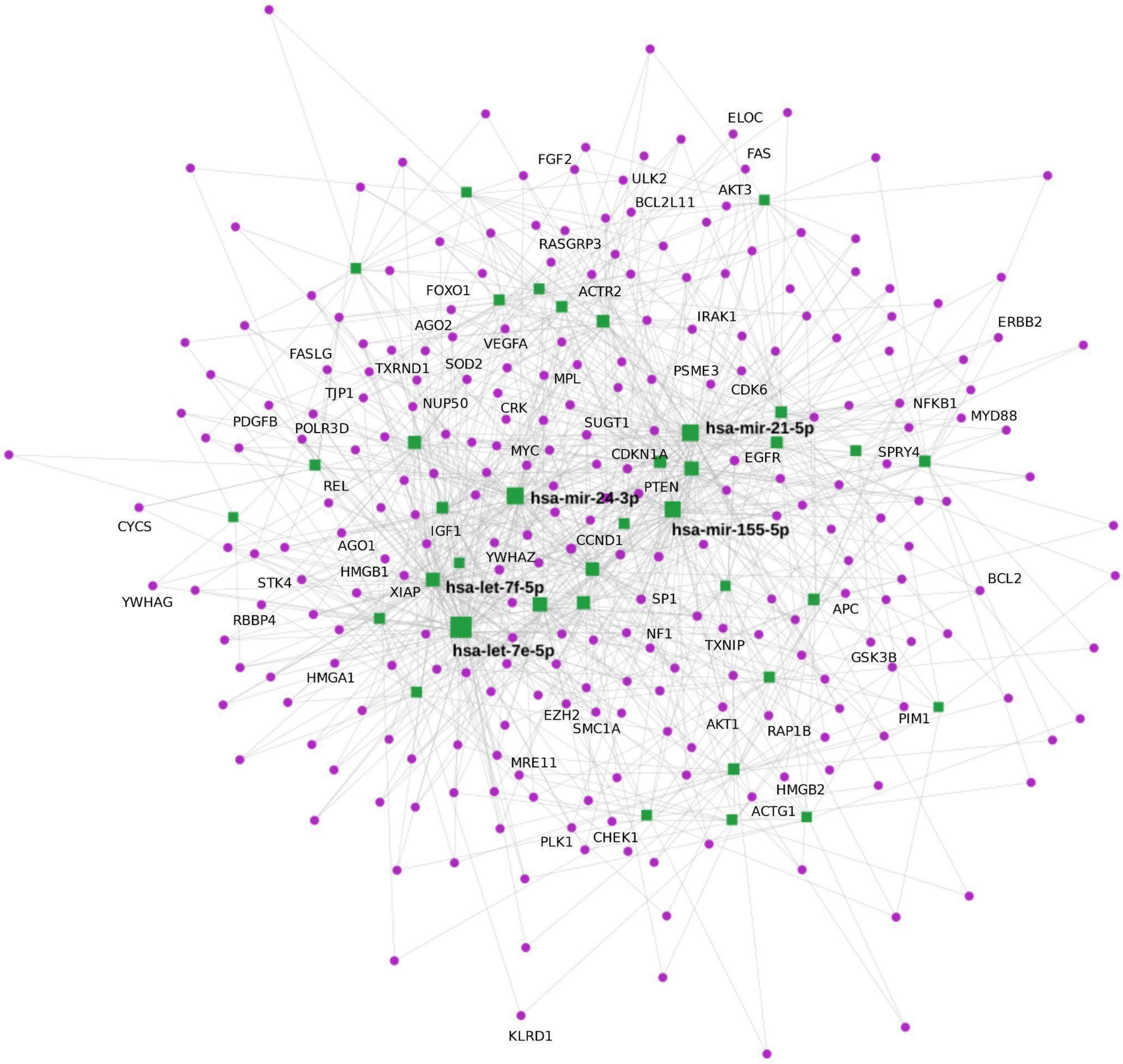

Figure S9. A miRNA-gene interaction network built upon the list of the 43 upregulated miRNAs as seeds carried on with miRNet (*T. cruzi*-THP1). In the network, let-7e-5p, let-7f-5p, miR-24-3p, miR-21-5p and miR-155-5p are highlighted as important hubs (node degree>50). Nodes are colored in green and purple for miRNAs and genes, respectively. Labels of miRNA targets are shown for those genes related to cell cycle, apoptosis, PI3K/Akt signaling and innate immune response.

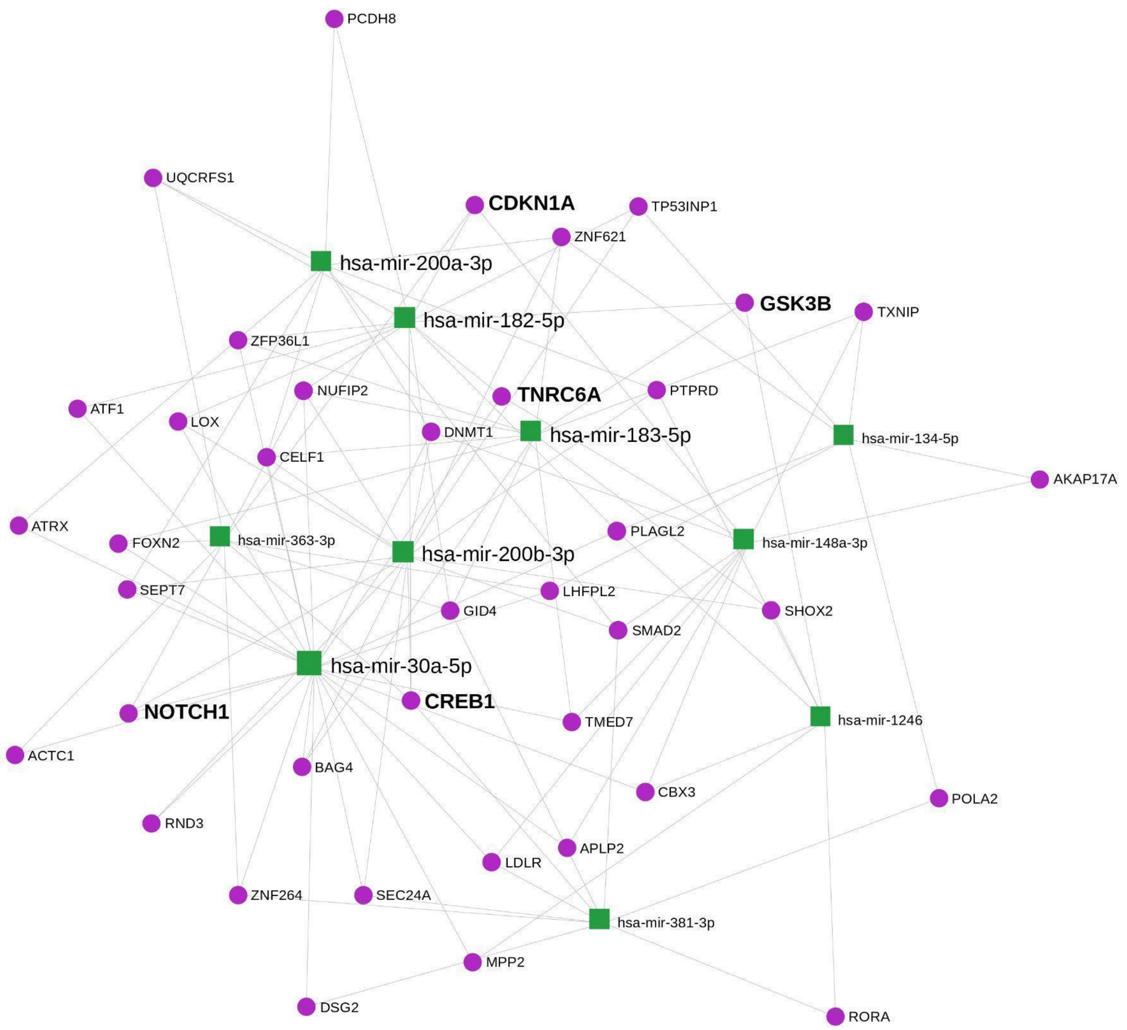

Figure S10. A miRNA-gene interaction network built upon the list of the 10 upregulated miRNAs as seeds carried on with miRNet (*T. cruzi*-cardiomyocytes). In the network, genes of interest NOTCH1, CREB1, GSK3B, CDKN1A and TNRC6A are highlighted in bold. Nodes are colored in green and purple for miRNAs and genes, respectively.
